# Adaptation via pleiotropy and linkage: Association mapping reveals a complex genetic architecture within the stickleback *Eda* locus

**DOI:** 10.1002/evl3.175

**Published:** 2020-05-27

**Authors:** Sophie L. Archambeault, Luis R. Bärtschi, Aurélie D. Merminod, Catherine L. Peichel

**Affiliations:** ^1^ Institute of Ecology and Evolution University of Bern Bern 3012 Switzerland; ^2^ Graduate Program in Molecular and Cellular Biology University of Washington Seattle Washington 98195; ^3^ Divisions of Basic Sciences and Human Biology Fred Hutchinson Cancer Research Center Seattle Washington 98109

**Keywords:** Adaptation, association mapping, *Ectodysplasin* (*Eda*), *Gasterosteus aculeatus*, lateral line neuromasts, lateral plates, linkage, pleiotropy, threespine stickleback

## Abstract

Genomic mapping of the loci associated with phenotypic evolution has revealed genomic “hotspots,” or regions of the genome that control multiple phenotypic traits. This clustering of loci has important implications for the speed and maintenance of adaptation and could be due to pleiotropic effects of a single mutation or tight genetic linkage of multiple causative mutations affecting different traits. The threespine stickleback (*Gasterosteus aculeatus*) is a powerful model for the study of adaptive evolution because the marine ecotype has repeatedly adapted to freshwater environments across the northern hemisphere in the last 12,000 years. Freshwater ecotypes have repeatedly fixed a 16 kilobase haplotype on chromosome IV that contains *Ectodysplasin* (*Eda*), a gene known to affect multiple traits, including defensive armor plates, lateral line sensory hair cells, and schooling behavior. Many additional traits have previously been mapped to a larger region of chromosome IV that encompasses the *Eda* freshwater haplotype. To identify which of these traits specifically map to this adaptive haplotype, we made crosses of rare marine fish heterozygous for the freshwater haplotype in an otherwise marine genetic background. Further, we performed fine‐scale association mapping in a fully interbreeding, polymorphic population of freshwater stickleback to disentangle the effects of pleiotropy and linkage on the phenotypes affected by this haplotype. Although we find evidence that linked mutations have small effects on a few phenotypes, a small 1.4‐kb region within the first intron of *Eda* has large effects on three phenotypic traits: lateral plate count, and both the number and patterning of the posterior lateral line neuromasts. Thus, the *Eda* haplotype is a hotspot of adaptation in stickleback due to both a small, pleiotropic region affecting multiple traits as well as multiple linked mutations affecting additional traits.

**Impact Summary**

When organisms adapt to new habitats, they often encounter a suite of environmental differences, including abiotic factors such as temperature or salinity, and biotic factors such as predator, prey, and parasite communities. Rapid adaptation to many different aspects of new environments can therefore be facilitated when the genetic changes (i.e., mutations) that contribute to adaptation in a suite of phenotypic traits are inherited together. This can occur when the same mutation leads to changes in many traits, known as pleiotropy, or when multiple mutations are located close to each other in the genome and are therefore inherited together, known as linkage. Although many studies have identified regions of the genome that underlie variation in suites of adaptive traits, whether these regions contain a single pleiotropic mutation or linked mutations is mostly unknown. Here, we use genetic mapping approaches in natural populations to dissect a 16‐kb genomic region that has previously been shown to be genetically differentiated between marine and freshwater threespine stickleback fish. This 16‐kb region contains a gene called *Ectodysplasin* (*Eda*), which is known to control differences in the number of bony lateral plates and in the lateral line neurosensory system between marine and freshwater sticklebacks. Here, we demonstrate that these phenotypic differences are due to the pleiotropic effects of a 1.4‐kb region within the first intron of the *Eda* gene. However, we also find that additional linked mutations modify the lateral plate and lateral line phenotypes and have minor effects on other phenotypes that differ between marine and freshwater sticklebacks. Thus, both linkage and pleiotropy contribute to rapid adaptation in this system. Furthermore, selection for linkage between these mutations is likely maintaining genetic differentiation between marine and freshwater sticklebacks across the 16‐kb region in the face of gene flow between them.

Adaptation to divergent environments is often associated with changes in many traits (Darwin 1859; Fisher 1930; Orr 2000). For example, when a marine fish colonizes freshwater, it not only encounters a new abiotic environment, but also a new biotic environment with different predators, prey, and parasites. Thus, adaptation to freshwater is expected to involve a suite of morphological, behavioral, and physiological changes. Co‐inheritance of these suites of traits under selection in a particular environment is predicted to facilitate adaptation by reducing the production of unfit combinations of phenotypes (Charlesworth and Charlesworth 1979; Kirkpatrick and Barton 2006; Hoffmann and Rieseberg 2008; Schwander et al. 2014). Indeed, genetic mapping of phenotypic changes in systems of adaptive evolution has identified clustering of traits, such that multiple traits are affected by a single genomic region or gene (Hawthorne and Via 2001; Albertson et al. 2003; McKay et al. 2003; Bratteler et al. 2006; Hall et al. 2006; Scarcelli et al. 2007; Protas et al. 2008; Lowry and Willis 2010; Joron et al. 2011; Parnell et al. 2012; Yoshizawa et al. 2012; Friedman et al. 2015; Peichel and Marques 2017). In most cases, it is unknown whether these phenotypic hotspots are due to the effects of a single pleiotropic mutation; or multiple, linked causative mutations; or a combination of both (but see Carbone et al. 2006; Hermann et al. 2013; Kamberov et al. 2013; Linnen et al. 2013; Lee et al. 2017; Dong et al. 2018; Erickson et al. 2018; Nagy et al. 2018; Butelli et al. 2019). Given the longstanding goal of linking adaptive phenotypes to their underlying genotypes as well as the sources of selection on those phenotypes (Barrett and Hoekstra 2011), knowing if a single mutation is affecting multiple traits or if multiple, linked mutations within a pleiotropic gene or genomic region underlie adaptive traits is crucial to understanding and predicting the adaptability of populations.

Pleiotropy could facilitate rapid, adaptive evolution, or adaptation in the face of gene flow, if all or most of the phenotypic changes were beneficial in the new environment. However, the classical view is that pleiotropy is more likely to constrain adaptation because the probability that a mutation with beneficial effects on one trait has detrimental effects on other traits and overall fitness (i.e., antagonistic pleiotropy) is predicted to increase with the degree of pleiotropy, imposing a “cost of complexity” (Fisher 1930; Orr 2000; Otto 2004). Indeed, the pleiotropic effects of many developmental genes and disease mutations has led to the expectation that morphological evolution is more likely to occur through mutations that reduce pleiotropy, such as tissue‐specific regulatory mutations (Carroll 2008; Stern and Orgogozo 2008, but see Hoekstra and Coyne 2007). Empirical work has found support for this expectation by linking morphological evolution to mutations in modular or tissue‐specific enhancers (Rebeiz et al. 2009; Chan et al. 2010; Frankel et al. 2011; Wallbank et al. 2016), but recent work has found that mutations in regulatory DNA can also have pleiotropic effects (Nagy et al. 2018; Lewis et al. 2019; Ramaekers et al. 2019; Sabarís et al. 2019). Meta‐analyses of genome‐wide association studies report widespread pleiotropy of genomic loci (Boyle et al. 2017; Chesmore et al. 2018; Watanabe et al. 2019). However, data from gene knockout studies in yeast, nematodes, and mice as well as quantitative trait locus (QTL) mapping in mice find that most genes or QTL exhibit little or no pleiotropy, but loci that are pleiotropic show a positive correlation between the per trait effect sizes of mutations with the number of traits affected (Wagner et al. 2008; Wang et al. 2010). Together these findings suggest that the “cost of complexity” imposed by pleiotropy can be overcome by facilitating larger steps toward the fitness optimum. However, pleiotropy is often measured at the level of QTL or genomic regions, and the extent to which the pleiotropic effects of a single mutation contribute to adaptation remains unknown.

Linkage of multiple adaptive mutations has also been proposed as a mechanism to facilitate rapid adaptation, particularly in the face of gene flow (Charlesworth and Charlesworth 1979; Kirkpatrick and Barton 2006; Hoffmann and Rieseberg 2008; Yeaman and Whitlock 2011; Ortiz‐Barrientos et al. 2016). Consistent with the theory that there is selection for tight linkage between alleles that contribute to adaptation, genomic regions of low recombination, such as inversions, often harbor loci important for many different traits (Lowry and Willis 2010; Joron et al. 2011; Fishman et al. 2013; Hermann et al. 2013; Wang et al. 2013; Kunte et al. 2014; Küpper et al. 2016; Lamichhaney et al. 2016; Tuttle et al. 2016; Lee et al. 2017; Cocker et al. 2018; Westram et al. 2018). With a few exceptions (Hermann et al. 2013), the number and nature of the causative mutation(s) in these regions have not yet been identified.

Linkage and pleiotropy have different implications for the speed, acquisition, and maintenance of phenotypic effects during adaptive evolution. For example, linked mutations are likely acquired one at a time, which could require longer to gain multiple phenotypic effects than a single, pleiotropic mutation. Furthermore, in contrast to the phenotypic effects of pleiotropic mutations, the effects of linked mutations can be separated by mutation or recombination given enough time, lowering the long‐term maintenance of genetic correlations. Therefore, disentangling the roles of pleiotropy and linkage has important implications for our understanding of adaptation (Barrett and Hoekstra 2011).

## Rapid and Repeated Freshwater Colonization by Threespine Stickleback

The threespine stickleback (*Gasterosteus aculeatus*) has become a model system for studying the genetic basis of adaptive evolution (Kingsley and Peichel 2007; Peichel and Marques 2017). Across its Holarctic range, marine stickleback have repeatedly invaded and adapted to freshwater environments, which has resulted in the parallel evolution of many traits. For example, most freshwater populations have reduced defensive bony armor, including bony lateral plates and spines, and changes in feeding morphology, including reduction of gill raker length and number (Hagen and Gilbertson 1972; Hendry et al. 2013). This parallel phenotypic evolution is mirrored at the genetic level by parallel fixation of shared freshwater haplotypes (Hohenlohe et al. 2010; Jones et al. 2012; Terekhanova et al. 2014; Bassham et al. 2018). These freshwater haplotypes exist as standing genetic variation in marine populations that persists through ongoing migration of alleles to and from freshwater (Colosimo et al. 2005; Hohenlohe et al. 2010; Jones et al. 2012; Nelson and Cresko 2018). Importantly, these freshwater haplotypes often overlap with known QTL for traits that differ between marine and freshwater sticklebacks, suggesting that they are key for the rapid, parallel evolution of many traits in freshwater (Hohenlohe et al. 2010; Jones et al. 2012).

## The Role of the *Eda* Gene as a Major Pleiotropic Gene in Freshwater Adaptation

**Figure 1:**
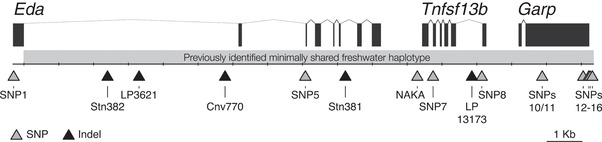
**The *Eda* haplotype contains three protein coding genes and 16 markers**. The minimal region shared by most low‐plated, freshwater populations of threespine stickleback is 16‐kilobase long (gray bar). It contains the majority of three protein coding genes: *Ectodysplasin* (*Eda*), *Tumor necrosis factor super‐family member 13b* (*Tnfsf13b*), and *Glycoprotein A rich protein* (*Garp*). Genotyping assays were designed for 17 markers (triangles) that distinguish the marine or “C” allele (associated with the completely plated phenotype) and the freshwater or “L” allele (associated with the low‐plated phenotype). These markers are a mix of SNPs and indels indicated by gray and black triangles, respectively, and are listed in Table S1. Note: Exon 1 of *Eda*, and therefore SNP1, is outside the minimally shared freshwater haplotype.

One of the strongest molecular signals of divergent selection between marine and freshwater stickleback genomes is in a 16‐kb region on chromosome IV (Jones et al. 2012). This 16‐kb region has a number of fixed sequence differences between the marine and freshwater haplotypes and contains three protein‐coding genes, *Ectodysplasin* (*Eda*), *Tumor necrosis factor super‐family member 13b* (*Tnfsf13b*), and *Glycoprotein A repetitions predominant* (*Garp*) (Fig. 1). The first of these genes, *Eda*, controls at least three traits: number of bony lateral plates, body position while schooling, and lateral line patterning (Colosimo et al. 2004; Colosimo et al. 2005; Wark et al. 2012; Greenwood et al. 2013; Mills et al. 2014; Greenwood et al. 2016). *Eda* is required for the development of epithelial appendages in vertebrates, including hair, teeth, scales, and lateral plates (Srivastava et al. 1997; Harris et al. 2008; Aman et al. 2018; Wucherpfennig et al. 2019). Protein sequence comparison and tissue‐specific expression level analysis suggest that the phenotypic differences driven by *Eda* are due to reduced expression of the freshwater allele (Colosimo et al. 2005; O'Brown et al. 2015). Consistent with this hypothesis, overexpression of *Eda* in freshwater sticklebacks partially recovers the ancestral marine phenotypes for number of lateral plates, body position while schooling, and lateral line patterning, confirming the pleiotropic role of *Eda* in phenotypic evolution of freshwater stickleback (Colosimo et al. 2005; Mills et al. 2014; Greenwood et al. 2016).

Despite clear evidence for the role of *Eda* in these phenotypes, the causative mutation(s) are unknown. Compared to marine fish, there are four coding changes in *Eda* shared by North American low‐plated stickleback. However, a low‐plated Japanese population (NAKA stream population) lacking the North American amino acid changes fails to complement for lateral plate formation, suggesting that plate loss is due to a shared regulatory, not coding, change (Colosimo et al. 2005). Consistent with this model, allele‐specific expression analysis revealed a *cis‐*regulatory downregulation of the freshwater allele of *Eda* in F1 hybrid flank tissue (O'Brown et al. 2015). To identify the regulatory mutation leading to plate loss, O'Brown and colleagues (2015) leveraged the Japanese NAKA population to identify a single base‐pair mutation located within a regulatory element 3′ of the *Eda* gene that is shared by all sequenced, low‐plated fish. Hereafter, we will refer to this mutation as the NAKA SNP (single nucleotide polymorphism). Wild‐caught marine fish heterozygous only at the NAKA SNP are completely plated (O'Brown et al. 2015), yet it remains unknown whether this single SNP is necessary or sufficient in the homozygous state to cause plate loss, or if other mutations are involved. It is also unknown whether lateral plate, lateral line patterning, and schooling behavior changes are caused by a single pleiotropic mutation or by linked mutations.

In addition to the three known phenotypes affected by *Eda*, chromosome IV harbors QTL for more traits than expected by chance (Albert et al. 2008; Miller et al. 2014; Peichel and Marques 2017). Many of these QTL have confidence intervals that overlap with the *Eda* haplotype. Furthermore, there is no evidence of an inversion in this genomic region (Jones et al. 2012), which should allow us to disentangle the effects of individual mutations on phenotypes due to the presence of recombination across this genomic region. This “phenotypic hotspot” is therefore an interesting and important test case for disentangling the relative roles of pleiotropy and linkage during adaptive evolution. Here, we addressed two complementary questions: (1) how many and which traits map to the 16‐kb freshwater haplotype on a marine genomic background and (2) are these phenotypes affected by the same pleiotropic mutation, or by separate tightly linked mutations? To address the first question, we identified wild‐caught marine fish heterozygous for the freshwater *Eda* haplotype, made crosses between these heterozygous fish, and phenotyped and genotyped the offspring to determine which of the traits previously associated with QTL overlapping the *Eda* haplotype are specifically associated with this 16‐kb haplotype. To address the second question, we first tested whether the previously identified regulatory NAKA SNP is sufficient to generate the freshwater lateral plate and lateral line phenotypes in a marine genomic background. We also performed association mapping of these lateral plate and lateral line traits, as well as other phenotypes associated with QTL overlapping *Eda*, in a polymorphic, interbreeding freshwater population of stickleback in which recombination has occurred between the marine and freshwater *Eda* haplotypes.

## Methods

### ETHICS STATEMENT

Fish were collected under the Washington Department of Fish and Wildlife scientific collection permits 14–311b, 15–033, and 16–066. Animal care and handling protocols were approved by the Fred Hutchinson Cancer Research Center Institutional Animal Care and Use Committee (protocol 1575) or the Veterinary Service of the Department of Agriculture and Nature of the Canton of Bern (VTHa# BE4/16).

### PUGET SOUND FISH COLLECTIONS, CROSSES, AND CARE

To quantify the effects of the *Eda* haplotype on different phenotypes, we made crosses between multiple marine fish that were heterozygous carriers of the freshwater *Eda* haplotype. By using multiple wild‐caught marine carriers of the haplotype, phenotypic effects observed in multiple crosses can be attributed to the *Eda* haplotype rather than to freshwater alleles at other genomic loci that may be present in individual wild‐caught fish. Sufficient numbers of potential parents were obtained by collecting marine fish from Puget Sound, Washington, during two consecutive summers. In the summer of 2015, marine fish were caught in a midwater trawl during a multi‐day sampling trip in the Whidbey Basin and Bellingham Bay areas of Puget Sound, WA. In June 2016, marine fish were collected nearshore in a beach seine in full saltwater in Clam Bay, Puget Sound, near Manchester, WA, with the help of the Washington Department of Fish and Wildlife. Because the sampling location and habitats were different between the sampling years, it is possible that these two samples represent separate marine populations of Puget Sound stickleback. Fish were transported and housed in the stickleback facility at the Fred Hutchinson Cancer Research Center. Animals were kept in standard 29 gallon aquarium tanks, each aerated with an air stone and containing 0.35% saltwater (3.7 g·L^−1^ Instant Ocean sea salt, Instant Ocean Spectrum Brands, Inc., Blacksburg, VA, USA; 0.003 g·L^−1^ NaHCO3; 0.0003 g·L^−1^ Ca(OH)_2_). Water was filtered through an external filter (AquaClear Power Filter, Rolf C. Hagan Inc., Baie d'Urfé, Quebec, Canada). The rooms were programmed to mimic summer light conditions (16 h light:8 h dark) and maintained at approximately 17.5°C. Fish were kept at a density of up to 24 adults per tank and were fed live brine shrimp nauplii in the mornings and frozen *Mysis* shrimp in the afternoons.

**Figure 2:**
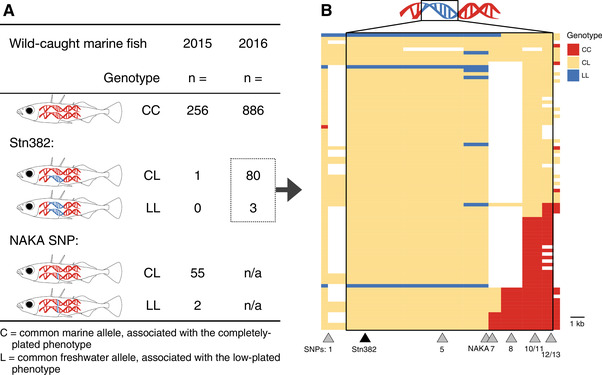
**Freshwater alleles persist in the marine population**. (A) Marine stickleback were collected and genotyped from Puget Sound in two consecutive summers, 2015 and 2016. In 2015, wild‐caught fish were sampled in midwater, genotyped first at the NAKA SNP, and subsequently at Stn382. The frequency of the L allele at the NAKA SNP was 9.4%, whereas the frequency of the full freshwater *Eda* haplotype was 0.16%. In 2016, fish were sampled nearshore, genotyped at Stn382, and the frequency of the L allele was 4.4%. (B) The carriers of the L allele at Stn382 from 2016 were genotyped at a subset of additional markers, and their genotypes are represented visually. Each row represents a single fish (*n* = 83), and the subset of markers genotyped within the haplotype (designated by the box) is shown below the plot. Triangles mark the physical location of SNP1 and the genotyped markers within the haplotype. Coloring representing the genotypes extends halfway to the next marker location. Additional markers on either side of the haplotype (Cnv767 and SNP19, ∼10 kb 5′ and 3′ of the region, respectively) were genotyped and are represented visually to the left of SNP1 and to the right of SNPs12/13, respectively. Missing data are in white.

In both 2015 and 2016, fish were individually marked with a combination of spine clipping and elastomer tagging, fin clipped, and genotyped following a HotSHOT DNA extraction (see “DNA EXTRACTIONS” section below). In 2015, fish were first genotyped at the NAKA SNP, an intergenic SNP 3′ of the *Eda* gene previously suggested to be involved in *Eda* regulation and plate reduction (O'Brown et al. 2015), and heterozygotes were then genotyped at Stn382, an indel polymorphism within the first intron of *Eda* that differentiates most marine from freshwater haplotypes (Colosimo et al. 2005) (Figs. 1 and 2; Table S1). Four crosses were made between fish that were heterozygous carriers of the freshwater allele at the NAKA SNP but that were homozygous for the marine allele at Stn382. One cross was made between a homozygous marine fish and the single heterozygous carrier of freshwater alleles at both the NAKA SNP and Stn382.

**Figure 3:**
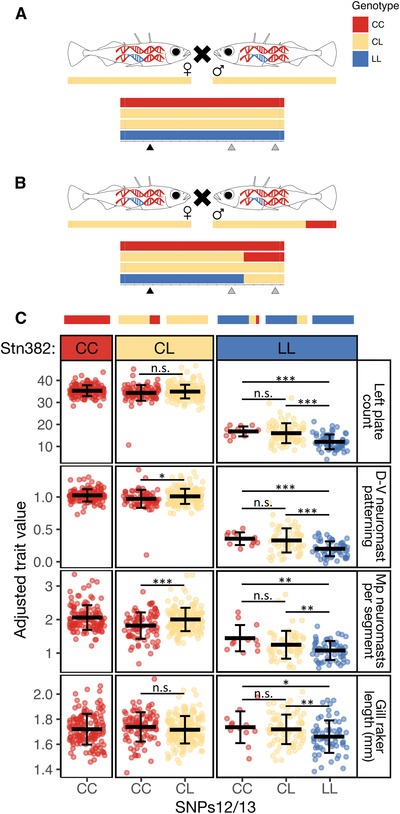
**Crosses between Puget Sound fish carrying different‐sized haplotypes reveal effects of freshwater alleles on multiple phenotypes**. (A) Schematic of the five crosses between heterozygous marine carriers of the full freshwater haplotype (CL genotype). (B) Schematic of the five crosses between one carrier of the full haplotype and one carrier of a short haplotype. In both (A) and (B), the possible genotypes of offspring are visualized below the parents, along with triangles marking the three markers at which offspring were genotyped—Stn382, NAKA SNP, and SNPs12/13. Not pictured is the single cross between two carriers of different short haplotypes, which produced some offspring with a tricolored haplotype, depicted in (C). (C) Trait values for four phenotypes are plotted by offspring genotypes at Stn382 and SNPs12/13. Representative haplotypes are drawn above the plots. Mean trait value ± SD are depicted by black lines and whiskers. Left plate count is strongly associated with genotype at Stn382 (LOD = 344; PVE = 93.6), and the C allele is dominant (CC and CL fish have similar trait values). However, SNPs12/13 are also associated with plate number in fish that are homozygous LL at Stn382 (fish LL at SNPs12/13 have fewer plates than fish CC and CL at SNPs12/13; *t*_151_ = –6.54, *P* = 4.4 × 10^–10^). The same pattern is observed in the dorsal‐ventral (D‐V) patterning of neuromasts and the number of neuromasts per segment in the Mp line. In contrast, gill raker length is not associated with genotype at Stn382, but it is associated with genotype at SNPs12/13 (LOD = 4.6; PVE = 3.7). Adjusted trait values were calculated by adding the residual trait value for each individual to the predicted trait value when all the covariates are equal (sex, standard length, and family). Significance levels: ^*^^*^^*^ *P* < 0.001; ^*^^*^ *P* < 0.01; ^*^ *P* < 0.05; *P* > 0.05 (not significant).

Given the higher frequency of NAKA heterozygotes to Stn382 heterozygotes in the 2015 sample (*n* = 55 and 1, respectively; Fig. 2), we reversed our genotyping strategy in 2016. Wild‐caught fish from 2016 were first genotyped at the marker Stn382 (Fig. 1; Table S1) to find heterozygous carriers of the freshwater *Eda* haplotype. Eleven crosses were made between fish that were heterozygous carriers of the freshwater allele at Stn382. Later genotyping (see “GENOTYPING ASSAYS” section below) revealed that five of these were crosses between carriers of the full freshwater *Eda* haplotype, five were crosses between carriers of a short freshwater haplotype with a carrier of a full freshwater haplotype, and one cross was between carriers of different short haplotypes (Fig. 3). Fish were considered heterozygous carriers of full freshwater haplotypes if they were heterozygous for all markers tested between Stn382 through SNP13, except the NAKA SNP. In contrast, short haplotypes varied in size, extending from Stn382 through somewhere between the NAKA SNP and SNP 11 (Fig. 2). In addition, an F2 cross was made between heterozygous F1 siblings from the single *Eda* full freshwater haplotype by homozygous F0 marine cross.

Offspring of these crosses were reared in the stickleback facility at the Fred Hutchinson Cancer Research Center under conditions similar to those for wild‐caught adults described above. The differences were that entire crosses were housed in a single tank and young fish were fed brine shrimp nauplii twice per day. The offspring of the 2016 crosses were shipped to the stickleback facility at the University of Bern, Switzerland, between the ages of 2 and 5 months (corresponding to 18‐35 mm standard length). Animals were housed in 100‐L tanks on a recirculating system. Conductivity (5.3 millisiemens/cm) and pH (7.5) were automatically monitored and maintained using saturated solutions of Instant Ocean sea salt (Instant Ocean Spectrum Brands, Inc., Blacksburg, VA, USA) and sodium bicarbonate (75 g·L^−1^). Lighting was programmed with 11 h full sunlight, 1 h sunrise, 1 h sunset, and a moon light for nighttime. Water temperature was maintained near 15°C. Crosses were housed in a single tank until they reached approximately 30 mm, and then they were split among multiple tanks to maintain approximately 45‐55 fish per tank. Young fish were fed brine shrimp nauplii twice per day, and adult fish were fed brine shrimp in the mornings and frozen *Mysis* shrimp three times per week in the afternoon. Fish were grown to about 2 years old before genotyping and phenotyping.

### LAKE WASHINGTON FISH COLLECTIONS AND CARE

To confirm the effects of the *Eda* haplotype on different genomic backgrounds and to fine map phenotypes within the haplotype, we used a polymorphic, interbreeding, freshwater population of stickleback; Lake Washington is a large freshwater lake near Seattle, WA that contains both completely and low‐plated sticklebacks. Genotype at Stn382 in the *Eda* haplotype explains 75.2% of the variation in plate phenotype in this population (Kitano et al. 2008). Adult fish were collected from different locations around Lake Washington between April 2015 and March 2016. Unbaited minnow traps were used to catch fish (*n* = 52) at Mercer Slough south of Bellevue, WA. A Merwin trap was set in Kenmore, WA, on the northern edge of Lake Washington (*n* = 136). Nighttime purse seining (*n* = 129) and trawling (*n* = 560) were conducted in the northern half of Lake Washington. The nearshore trapping (minnow and Merwin traps) was conducted in May, June, and July when adult sticklebacks are in nearshore habitat. The seining and trawling were used in October and March, respectively, when adult sticklebacks are found offshore. We sampled fish from a subset of locations around the lake because collections from 2005 (Kitano et al. 2008; locations 2, 4, 7, and 9) demonstrated that although fish from different locations have varying degrees of marine ancestry, there is no correlation between genotypes at neutral markers and plate phenotype. Based on this previous work, we expected low‐ and completely plated fish to be interbreeding, and the frequency of the freshwater *Eda* allele to be ∼40%.

Animals were housed in the stickleback facility at the Fred Hutchinson Cancer Research Center as described above for Puget Sound wild‐caught fish. Fish were kept in the lab for between 0 and 57 days before phenotyping.

### PHENOTYPING

To disentangle the relative roles of pleiotropy and linkage within the highly divergent marine‐freshwater haplotype on chromosome IV, we focused on phenotypes that had previously mapped to chromosome IV in QTL crosses between marine and freshwater stickleback (Peichel and Marques 2017). To fully analyze the geometric morphometric landmarks and lateral line neuromast counts (see below), we also included traits in these categories that were not previously mapped to chromosome IV (Table S2). For logistical reasons, we did not measure any aspect of schooling behavior, which has previously been associated with variation in *Eda* (Greenwood et al. 2016).

### PHENOTYPING: LATERAL LINE NEUROMASTS

The Puget Sound NAKA SNP crosses (*n* = 86 individuals) and a subset of Lake Washington fish (244 of 768) were stained to visualize lateral line neuromasts using the fluorescent vital dye 2‐(4‐(dimethylamino)styrl)‐N‐ethylpyridinium iodide (DASPEI; Invitrogen/Molecular Probes, Carlsbad, CA) as described in Wark et al. (2012). Neuromasts were visualized and counted as described below using a Leica dissecting scope with fluorescent light and a FITC filter set (Leica Microsystems Inc., Banncokburn, IL, USA). After staining, fish were euthanized with a lethal dose of MS‐222, fins were clipped and saved in 95% ethanol for DNA extraction, and fish were placed in a T‐Sac Tea Filter Bag (Magic Teafit, Columbus, OH, USA) with a waterproof, unique ID tag. Fish were then stored in Mason jars in 10% buffered formalin for at least one week before staining with Alizarin red to visualize bony structures, as described by Peichel et al. (2001).

DASPEI staining is time‐intensive and variable because each fish is stained and screened live, and the quality of staining can abruptly change if the fish is anesthetized for too long. We therefore adapted a method of neuromast staining using alkaline phosphatase to allow for the bulk preservation, staining, and storage of fish, plus reliable and convenient phenotyping (adapted from Villablanca et al. 2006). This method was used to phenotype the Puget Sound *Eda* haplotype crosses (*n* = 498) and the remaining Lake Washington fish (524 of 768). Fish were first placed in aerated epinephrine for 10 min to contract their melanophores (0.07 g·L^−1^ epinephrine in fish water). Fish were transferred to a lethal dose of MS‐222 and left until all operculum movement had stopped for 4 min. Standard length was recorded, and fins were clipped and placed in 95% ethanol for DNA extraction. Fish bodies were then placed with a waterproof ID tag in custom staining chambers (Fig. S1), and submerged in freshly made, cold 4% paraformaldehyde. Fish were kept at 4°C for 24‐36 h, then rinsed three times for 10 min in PBS and returned to 4°C for 12‐72 h. On the day of neuromast staining, fresh coloration buffer was made (0.1 M NaCl; 0.1 M Tris‐HCl, pH 9.5; 0.05 M MgCl_2_; 0.1% Tween‐20 in reverse osmosis water). Fish were rinsed in coloration buffer for 20 min, then submerged in half‐strength NBT/BCIP solution (0.225% NBT, 0.175% BCIP in coloration buffer; NBT/BCIP from Promega Corporation, Madison, WI, USA). Staining was carried out in the dark for 3‐4 hours on a rotational shaker. The preopercular and infraorbital lateral lines were checked periodically on a dissecting microscope to assess staining, as these lines are easy to find and often stain well. High‐quality staining produces a circle around the outer edge of the neuromast, and sometimes a line or dot in the middle of the neuromast (Fig. S1). Once this pattern is dark, staining can be terminated by a 10‐min rinse in PBS, followed by two methanol rinses to intensify staining (30 min in 25:75 methanol:PBTw (0.1% Tween in PBS), 20 min in 43:57 methanol:PBTw). Fish were then rinsed twice for 10 min in PBS and could be stored at 4°C for up to 3 days in PBS. Fish could be screened at this point, but we found it easier to screen them after Alizarin red staining. Fish were transferred to tea bags and stored in 10% buffered formalin at room temperature before Alizarin red staining as described previously (Peichel et al. 2001). Fish stained with alkaline phosphatase/Alizarin red were submerged in water and gently brushed with a paintbrush to remove background alkaline phosphatase stain on the bones surrounding the neuromasts. Neuromasts were viewed and counted on a Leica dissecting scope using visible light as described below (Leica Microsystems Inc., Bannockburn, IL, USA).

Neuromasts were counted in the 12 stickleback lateral lines, as described previously (Wark and Peichel 2010; Wark et al. 2012) (Fig. S1; Table S2). In addition to counting the neuromasts in the anterior and posterior main trunk lateral lines (Ma and Mp, respectively), the neuromast pattern was drawn for each plate and/or body segment. These drawings were later used to count neuromasts in the Ma and Mp lines and to quantify the dorsal‐ventral (D‐V) neuromast patterning. Because staining quality varied along the anterior‐posterior axis and some segments lacked neuromasts due to poor staining quality, the anterior and posterior trunk line (Ma and Mp) neuromast counts were averaged per body segment with stained neuromasts. Additionally, each body segment of the Mp line was phenotyped as having a midline neuromast pattern (with neuromasts lying directly on the midline) or a dorsal‐ventral neuromast pattern with at least one neuromast off the midline. This trait is reported as the fraction of segments displaying the dorsal‐ventral pattern (D‐V patterned segments).

### PHENOTYPING: METRICS AND MERISTICS

Meristic and metric traits were scored under a Leica dissecting scope. Meristic traits included plate count on the left side of the fish, anal fin ray number, and dorsal fin ray number (Table S2). Metric traits were measured using S_Cal PRO IP67 digital calipers (Sylvac SA, Crissier, Switzerland), and included standard length, pelvic girdle length, ectocoracoid length, spine lengths (first, second, and third dorsal spines; anal spine; and left pelvic spine), middle gill raker length, and supraoccipital notch length (Table S2). To expose the gill rakers for measurement, the branchiostegal rays were cut and the operculum was peeled up to expose the branchial bones and gill rakers. The length of the middle anterior facing gill raker on the first gill arch was measured with the digital calipers.

### PHENOTYPING: BRANCHIAL TRAITS

Branchial skeletons from 98 Puget Sound cross offspring and 76 wild‐caught Lake Washington fish were dissected and mounted following the protocol of Ellis and Miller (2016). Three categories of branchial traits were phenotyped following Miller et al. (2014): pharyngeal tooth counts, gill raker counts, and branchial bone lengths. The counting of pharyngeal teeth on the right two dorsal and single ventral toothplates was done under a binocular microscope using a black background to increase contrast. Teeth were counted between two and eight times by the same researcher to improve precision. Anterior‐facing gill rakers were counted on the first left branchial arch following Miller et al. (2014). The fourth ceratobranchial bone on the left side was measured using digital calipers on the mounted branchial skeleton.

### PHENOTYPING: MORPHOMETRICS

Fish were pinned to a dissecting mat for consistency between fish. Insect pins were inserted at landmarks 2, 8, 23, and 29 corresponding to the posterior insertion of the last anal fin ray, the anterior edge of the ectocoracoid, the supraoccipital notch, and the posterior insertion of the last dorsal fin ray, respectively (Fig. S2). Photos were taken of each fish with a 1‐cm reference scale in the picture. Photos were converted into .tps files using the *tps.util* program. Twenty‐nine landmarks were placed on each fish using *tps.dig2* (Fig. S2; Table S2). If landmarks could not be placed without doubt, placeholder landmarks were used and these fish were removed from the data before the morphometric analysis. Landmark coordinates were scaled and rotated using the *geomorph* package in R (https://cran.r-project.org/package=geomorph), and the scaled and rotated *X* and *Y* coordinate values were used as trait values in further analyses. Two linear measurements were extracted from the scaled landmark positions: body depth and maxilla length. Body depth was calculated as the distance between landmarks 6 and 25, and maxilla length as the distance between landmarks 18 and 16 (Fig. S2).

### DNA EXTRACTIONS

DNA from fin tissue of wild‐caught Puget Sound fish was extracted using a modified HotSHOT DNA extraction method (Meeker et al. 2007). Fin tissue was placed directly into 30 μL of 50 mM NaOH, digested at 95°C for 30 min, cooled on ice, and then pH neutralized with 8 μL of 1 mM TrisHCl. Samples were spun down for 10 min and 0.5 μL of the supernatant was used in 5 or 10 μL PCR reactions.

Fin tissue from Puget Sound crosses and Lake Washington fish were kept in 95% ethanol for DNA extractions. DNA was either extracted following a standard phenol‐chloroform protocol or using the Wizard^®^ SV 96 Genomic DNA Purification System (Promega, Madison, WI, USA). Following resuspension in TE buffer, DNA was quantified on a NanoDrop and diluted to 10 ng/μL in water for genotyping assays.

### GENOTYPING ASSAYS

Wild‐caught Puget Sound and Lake Washington fish were initially genotyped using PCR‐based methods at a subset of known SNPs and indels across the 16‐kb region (Stn382, SNP5, NAKA SNP, SNPs10/11, and SNPs12‐16). Puget Sound fish were additionally genotyped at two flanking markers outside the 16‐kb region (Cnv767 and SNP19, ∼10 kb 5′ and 3′ of the 16‐kb region, respectively) (Colosimo et al. 2005; Lowe et al. 2018) (See Table S1 for details of PCR primers and genotyping assays). Fish were also genotyped for a marker in the 3′ UTR (untranslated region) of the isocitrate dehydrogenase gene (*Idh*) that distinguishes males from females (Peichel et al. 2004). Puget Sound F1 offspring from parents carrying various‐sized freshwater *Eda* haplotypes were genotyped at two markers: Stn382 and SNPs12/13 (Table S1). This allowed us to distinguish between offspring carrying a long haplotype from those carrying a short haplotype. Puget Sound F1 offspring of parents heterozygous only at the NAKA SNP were genotyped only at the NAKA SNP because there was no known variation at any other marker in the haplotype in the F0 parents.

Following initial genotyping at this subset of known polymorphisms, additional genotyping was performed on recombinant Lake Washington fish to fine‐map their recombination breakpoints and increase mapping resolution. Both known and novel polymorphisms were used in fine mapping (SNP1, LP3621, Cnv770, Stn381, SNP7, LP13173, and SNP8) (Table S1). Novel putative shared polymorphisms were selected using three datasets. First, published genomic and BAC sequences from one saltwater (Salmon River marine fish, GenBank: AC144489.2) and two freshwater individuals (Paxton benthic and Bear Paw fish, GenBank: AY897589.1 and AANH00000000.1, respectively) were aligned in Geneious version R9 (http://www.geneious.com; Kearse et al. 2012), and putative SNPs and indels were identified. Next, the sequencing data from Jones et al. (2012) were used to confirm the usefulness of SNPs or indels across multiple freshwater and marine populations because it contains low‐coverage whole genome sequences from 10 paired freshwater and saltwater populations (sticklebrowser.stanford.edu). A putative SNP was confirmed if the same freshwater allele was found in all freshwater populations with data and not in any marine populations. We also looked at insertions or deletions (indels) on the freshwater haplotype, as these may play an important role in phenotypic evolution (Lowe et al. 2018). The raw reads from the Jones dataset were used to confirm putative indels. For each putative indel, consensus marine and freshwater query sequences were created from the alignment of published genomes and BAC clones described above. Next, raw sequencing reads from the Jones dataset were separated into marine and freshwater databases and searched for exact matches to the query sequences. A putative indel was confirmed if the freshwater query accumulated multiple matches to the freshwater database and the marine query accumulated multiple matches to the marine database. This much reduced set of putative haplotype indels and SNPs were then used to design additional genotyping assays for screening recombinant fish (LP3621, Cnv770, and LP13173) (Table S1).

### CURATION OF GENOTYPE DATA FOR ASSOCIATION MAPPING

**Figure 4:**
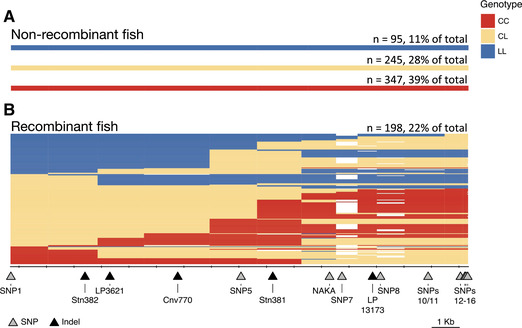
**Visual genotypes of Lake Washington fish reveal historical recombination events within the *Eda* haplotype**. (A) Wild‐caught adult stickleback from Lake Washington (*n* = 885) were genotyped at a subset of 16 SNPs or indels across the haplotype and SNP1. Most fish showed no evidence of recombination within the 16‐kb haplotype. (B) There is evidence of at least one historical recombination event within the haplotype in 198, or 22%, of the fish. The markers are depicted as triangles at their physical location relative to the haplotype and labeled at the bottom of the figure. Tick marks start at the beginning of intron 1 of *Eda* (the presumed start of the haplotype) and are spaced every 1000 bases. Genotypes are represented visually as CC (homozygous for the completely plated allele), CL (heterozygous), or LL (homozygous for the low‐plated, typically freshwater, allele), and extend halfway between each marker position. Missing genotypes are in white. The number of fish (*n*) and % of fish for each category of haplotype are listed on the right.

Puget Sound offspring were successfully genotyped at the chosen informative markers for all fish. For the Lake Washington fish, missing genotypes were dealt with in the following manner. Missing genotypes were imputed if the genotypes at both genotyped flanking markers were the same, and no genotyped sample within the dataset had an incongruous genotype at that marker (with the assumption of no double recombination events). In addition, two or three missing genotypes in a row were filled in following the same guidelines as for a single missing marker. Figures 4 and S3 show the genotypes at each marker (with missing data in white) prior to imputation. The following numbers for each marker are the numbers of successfully genotyped samples/imputed genotypes SNP1: 884/0, Stn382: 883/2, LP3621: 860/18, Cnv770: 852/0, SNP5: 874/0, Stn381: 863/21, NAKA SNP: 884/0, SNP7: 386/498, LP13173: 838/46, SNP8: 706/178, SNPs10/11: 877/7, and SNP13: 885/0. Our method of imputing missing data could lead to incorrect genotype information; therefore, a second trimmed genotype file was created in which all missing genotypes were deleted. This mostly involved deleting individual fish from the analysis; however, all genotypes for SNP7 and SNP8 were deleted because they had missing data for over half of the fish. We report the association mapping results using the trimmed genotype file because it produced qualitatively the same results as the filled genotype file but is more conservative.

### ANALYSES

Puget Sound crosses and Lake Washington wild‐caught fish were analyzed in largely the same way and differences will be noted below. Outliers were assessed in two ways and then removed. First, prior to analyses, data were explored by eye for categories of fish that should be removed. For example, for lateral line traits, fish with poor staining quality (assessed subjectively during phenotyping on a scale of 1‐7) were removed from analyses (*n* = 161 in Lake Washington; *n* = 86 in Puget Sound). For non‐lateral line traits: the following samples were removed from Lake Washington analyses: June and July males (*n* = 6 and *n* = 3, respectively), October samples larger than 65 mm (*n* = 9; these fish are likely a year older than all other fish, because they are bigger than reproductive summer fish, and much bigger than October fish), and May fish less than 50 mm (*n* = 1). Second, trait values >4 standard deviations from the mean trait value were also removed prior to analysis of the Lake Washington and Puget Sound datasets (between 0 and 2 samples per trait were removed totaling 30 trait values across all analyses). The number of fish analyzed for each phenotype and dataset is provided in Table S2.

To assess whether genotype is significantly associated with phenotype in the Puget Sound crosses and the wild‐caught Lake Washington fish, linear models representing the null and alternate hypotheses were compared. The null model included presumed covariates. The alternate hypothesis included covariates plus the genotype at the focal marker (i.e., Stn382). The models were compared using both a Chi‐square test and by calculating the log‐odds likelihood ratio (LOD) of the two models following the formula:

$$\mathrm{LOD}\;=\;\frac{n}{2}\times \log_{10}\left(\frac{RSS0}{RSS1}\right),$$

where *n* is the number of samples and *RSS*0 and *RSS*1 are the residual sums of squares of the null and alternate models, respectively (Broman and Sen 2009). We then calculated the percent of phenotypic variation explained (PVE) by each marker using the formula (Broman and Sen 2009):

$$\mathrm{PVE}\;=\;1-10^{\left(\frac{-2\;\times \;\mathrm{LOD}}{n}\right)}.$$

Using this method, we asked how much additional phenotypic variation is explained by genotype at the focal marker, after accounting for possible covariates. Variables that possibly contributed to variation in the raw trait values include biological variables such as sex, standard length, and family; collection variables including month of collection, lab tank housed in, and days kept in tank before staining; and method of staining (DASPEI vs. alkaline phosphatase staining for lateral line neuromasts) (Table S2). Therefore, covariates in the Puget Sound cross analyses included family, standard length, sex, and interactions between standard length and family, and sex and family. Covariates in the Lake Washington fine mapping varied by trait. Geometric morphometric data were already corrected for size, so covariates were sex and month of collection, plus their interaction. Meristic and metric traits were corrected for size, sex, and month of collection, plus interactions between sex and month and between size and month. These interactions allow for changes between sex or size and trait value that could change across life stages, such as pre‐reproductive and reproductive fish collected in October and June, respectively. Because lateral line traits were correlated with staining quality, which was susceptible to holding tank and staining method, covariates were sex, size, month of collection, tank, staining method, days held in tank, tank, and staining quality. Interactions between sex and month, and size and month were also included.

Significance of LOD values was assessed via permutation testing. For the Puget Sound crosses, we stratified the data for the permutation testing to account for possible differences between the size of the haplotype in each cross. Genotypes were only permuted within families with roughly the same haplotype sizes. The Lake Washington data were stratified by collection month to adjust for nonrandom differences in allele frequencies between collections. A total of 5000 permutations were performed. *P*‐values for each LOD score were calculated using these permutations, and the *P*‐value threshold was adjusted for multiple comparisons using a Bonferroni correction for number of traits tested (0.05/# of traits).

Power analyses were conducted using the *detectable()* function in the qtlDesign package in R (Sen et al. 2007). The detectable PVEs at 90% power were calculated for each trait in both mapping populations and were compared with previously published PVE for detected QTL on chromosome IV (Table S2). Inputs for the function included number of fish, error variance, and the LOD threshold. The error variance, *σ* ^2^, was calculated using the formula:

$$\sigma^{2}=\frac{RSS1}{\left(n-p-1\right)},$$

where *RSS*1 is the residual sum of squares of the alternate model, *n* is the number of samples, and *p* is the number of parameters in the alternate model including covariates and genotype at the focal marker. The LOD threshold and PVE were extracted from the 5000 permutations at the Bonferroni‐corrected level of significance.

## Results

### FRESHWATER HAPLOTYPE SIZE IS VARIABLE IN MARINE FISH

Genotyping of marine fish revealed that some fish in the Puget Sound marine populations are heterozygous carriers of freshwater *Eda* haplotypes (Fig. 2). Here, freshwater alleles are designated “L,” due to their association with the low‐plated phenotype, whereas alleles typically shared by marine fish are designated as “C” for completely plated (Barrett et al. 2008). Previous collections of completely plated marine fish estimated the frequency of the L allele at Stn381 (an indel in intron 6 of *Eda*) to be 3.8% and 0.2% in populations from California and British Columbia, respectively (Colosimo et al. 2005). The frequency of the L allele at Stn382 (an indel in intron 1 of *Eda*) in our nearshore 2016 collection was 4.4% (*n* = 86 of 1938 chromosomes). Further genotyping across the haplotype region revealed that most fish carried the full 16‐kb freshwater haplotype, but some fish carried a shorter version (47 “full‐L” haplotypes, 39 “short‐L” haplotypes; Fig. 2). These shorter versions of the haplotype varied in size from nearly the full 16‐kb region to somewhere between 10 and 12 kb, down to possibly just a single base pair at the NAKA SNP. Similar genotyping of the 2015 Puget Sound fish caught in a midwater trawl found frequencies of the full‐L haplotype to be 0.16% and at the NAKA SNP to be 9.4% (*n* = 1 and 59 of 628 chromosomes, respectively). In comparison, previous sampling of completely plated marine fish in Alaska estimated the frequency of the L allele at the NAKA SNP to be 1.5% (O'Brown et al. 2015).

### PUGET SOUND MARINE CROSSES IDENTIFIED FOUR TRAITS THAT MAP TO THE *Eda* HAPLOTYPE

In the offspring of crosses between heterozygous carriers of the *Eda* full‐L and short‐L freshwater haplotypes, genotype at the *Eda* haplotype was significantly associated with four of the 17 measured phenotypes: left plate count (LOD = 344; PVE = 93.6), dorsal‐ventral (D‐V) neuromast patterning (LOD = 292; PVE = 93.3), Mp neuromasts per segment (LOD = 77.1; PVE = 51.0), and gill raker length (LOD = 4.65; PVE = 3.68) (Fig. 3; Tables 1 and S2). The C allele is dominant for these traits; heterozygous “CL” and homozygous “CC” fish have similar phenotypes and differ from “LL” fish (Fig. 3). No other measured traits that have been previously mapped to QTL on chromosome IV were significantly associated with the *Eda* haplotype in these crosses. We had low power to detect associations that explained less than 18% of the variance in branchial traits due to the low numbers of fish measured. For all other measured traits, including dorsal and pelvic spine lengths, pelvic girdle length, or ectocoracoid length, we still had high power (90%) to detect associations that explained as little as 3.4% of the phenotypic variance (Table S2).

**Table 1:** **Summary of traits that mapped to the *Eda* haplotype in either the Puget Sound crosses or the Lake Washington wild‐caught population**. The traits that mapped significantly in each mapping population are listed (no measured traits reached significance in the NAKA crosses) along with the number of fish (*n*), the marker with the highest LOD score, the percent variance explained (PVE) by genotype, the *P*‐value from the ANOVA after accounting for covariates, and the adjusted *P*‐value threshold that was used to determine significance after accounting for multiple comparisons (Bonferroni correction)

Mapping population	Trait type	Trait name	*n*	Marker	LOD	PVE	*P*‐value	Adjusted *P*‐value threshold
Puget Sound crosses	Lateral line	Mp neuromasts per segment	497	Stn382	77.1	51.0	0.0000	0.0029
	Lateral line	Dorsal‐ventral (D‐V) neuromast patterning	498	Stn382	292.3	93.3	0.0000	0.0029
	Meristic	Left plate count	578	Stn382	344.3	93.6	0.0000	0.0029
	Metric	Middle gill raker length	571	SNPs12/13	4.6	3.7	0.0002	0.0029
Lake Washington wild‐caught	Lateral line	Mp neuromasts per segment	460	Cnv770	30.7	26.5	0.0000	0.0005
	Lateral line	Dorsal‐ventral (D‐V) neuromast patterning	460	Cnv770	113.4	67.9	0.0000	0.0005
	Meristic	Left plate count	696	Cnv770	218.3	76.4	0.0000	0.0005
	Morphometric	Posterior extent of operculum (Y10)	545	Stn381	3.9	3.3	0.0000	0.0005
	Morphometric	Posterior extent of maxilla (X16)	545	SNP5	3.0	2.5	0.0004	0.0005
	Morphometric	Anterior extent of maxilla (Y18)	545	Stn381	3.0	2.5	0.0004	0.0005

Crosses between heterozygous carriers of different‐sized freshwater haplotypes allowed us to assess which part of the haplotype contains the causative variants by comparing the average phenotypic residuals of offspring with different combinations of haplotypes. We found that gill raker length is not significantly associated with Stn382 but is associated with SNPs12/13 (LOD = 4.65; Fig. 3; Table S2). In contrast, the variation in plates and the two lateral line traits is more strongly associated with Stn382 than SNPs12/13 (i.e., LOD = 344 at Stn382 vs. 114 at SNPs12/13 for plates; Table S2).

### MULTIPLE MUTATIONS WITHIN THE 16‐kb HAPLOTYPE AFFECT LATERAL PLATE AND LATERAL LINE TRAITS

As described above, genotype at Stn382 is highly correlated with variation in plate number, neuromast patterning, and neuromast number in the Puget Sound marine crosses. However, additional variation in these three traits is associated with genotype at SNPs12/13 (Fig. 3; Table S3). For example, fish that are LL at both Stn382 and SNPs12/13 have significantly fewer D‐V patterned segments, Mp neuromasts per segment, and plates than fish that are LL at Stn382 and CL at SNPs12/13. These data suggest that there are at least two mutations within the haplotype that affect these three traits. The smaller effect mutation acts either epistatically with the larger effect mutation (an effect of SNPs12/13 is only observed when the genotype at Stn382 is LL), or the freshwater allele of the mutation near SNPs12/13 is recessive to the marine allele.

### THE NAKA SNP IS NOT SUFFICIENT TO CAUSE FRESHWATER LATERAL PLATE OR LATERAL LINE PHENOTYPES

**Figure 5:**
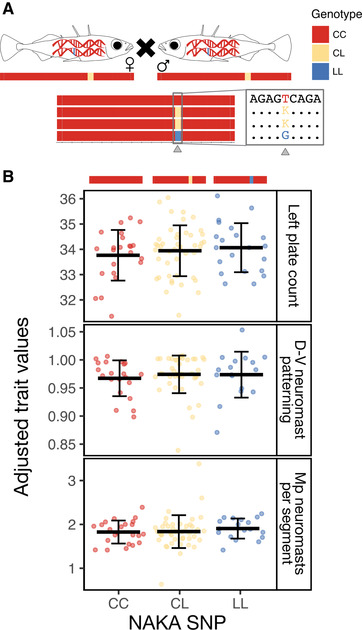
**The NAKA SNP is not sufficient to cause variation in traits**. (A) Schematic of crosses between heterozygous marine carriers of the NAKA SNP (CL genotype). Possible offspring genotypes are visualized below the parents, along with a triangle marking the NAKA SNP. The box contains the DNA sequence immediately surrounding the NAKA SNP. (B) Trait values for three phenotypes are plotted by offspring genotypes at the NAKA SNP. Representative genotypes are drawn above the plots. Mean trait value ± SD are depicted by black lines and whiskers. There is no association between genotype at the NAKA SNP and any of the three phenotypes—left plate count, dorsal‐ventral (D‐V) patterning of neuromasts or neuromasts per body segment along the posterior main trunk line (Mp). Adjusted trait values were calculated by adding the residual trait value for each individual to the predicted trait value when all the covariates are equal (sex, standard length, and family).

We next tested whether fish homozygous for the L allele at the NAKA SNP (contained within the short‐L haplotypes; Fig. 2) had significantly different trait values for plate number, as hypothesized by O'Brown et al. (2015), or neuromast number or neuromast patterning, given the strong correlation of these traits during development (Mills et al. 2014). In crosses between Puget Sound marine fish heterozygous only at the NAKA SNP, we found no significant effect of genotype on plate count, neuromast number, or neuromast pattern (Fig. 5; Table S2). These data are consistent with the finding that the two wild‐caught Puget Sound marine fish homozygous for the L allele only at the NAKA SNP were completely plated, whereas the three wild‐caught Puget Sound fish homozygous for the short‐L haplotype were low plated (Fig. 2).

### ASSOCIATION MAPPING OF TRAITS IN A FRESHWATER, POLYMORPHIC POPULATION OF STICKLEBACKS

To determine whether the genotypic correlations of lateral plates and lateral line traits are due to pleiotropic effects of a causative mutation(s) or due to tight genetic linkage of multiple causative mutations, we performed fine‐mapping in a polymorphic, interbreeding, freshwater population from Lake Washington in Seattle, WA (Kitano et al. 2008). Genotyping of 885 fish within the 16‐kb haplotype identified 198 fish (22%) with a historical recombination event within the 16‐kb haplotype (Figs. 4 and S3). These historical recombination events reduce linkage disequilibrium between the markers and enable association mapping between traits and genotypes at each marker within the haplotype.

**Figure 6:**
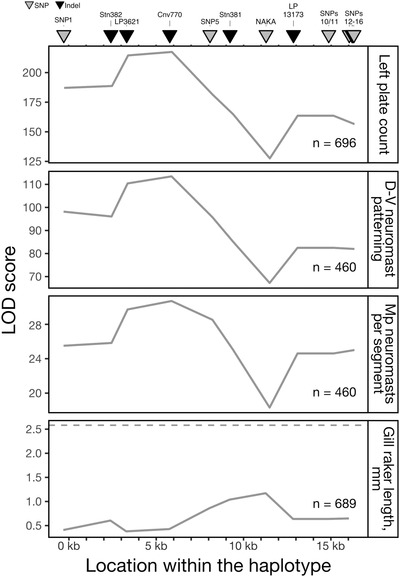
**Association mapping of traits in Lake Washington stickleback reveals a similar pattern of association shared by three traits**. The strength of association between each marker and each phenotype was calculated as a log odds likelihood (LOD) score compared with the model of no association between marker and phenotype. These LOD curves are plotted for four traits. Left plate count, dorsal‐ventral patterned segments, and Mp neuromasts per segment have significant LOD scores (LOD thresholds range from 2.7 to 2.9) and show very similar LOD curves, suggesting these traits are controlled by the same mutation(s). The LOD curve for gill raker length is shown, because this trait significantly mapped to the 3′ region (SNPs12‐13) of the haplotype in the Puget Sound crosses. However, as shown here, this trait does not significantly map to any marker within the haplotype in the Lake Washington population (LOD threshold = 2.6, dashed line).

In the Lake Washington mapping population, we again found that left plate count (LOD = 218; PVE = 76.4), dorsal‐ventral (D‐V) neuromast patterning (LOD = 113; PVE = 67.9), and Mp neuromasts per segment mapped to the *Eda* haplotype (LOD = 30.7; PVE = 26.5) (Fig. 6; Tables 1 and S2). In this population, we did not recover a significant correlation with gill raker length after correction for multiple comparisons (LOD = 1.17; PVE = 0.78). Some additional traits show an association but do not survive multiple correction, including anal spine length (LOD = 2.68; PVE = 1.77), left pelvic spine length (LOD = 1.94; PVE = 1.28), and pelvic girdle length (LOD = 2.75; PVE = 1.80). Despite high power to detect associations that explained as little as 3.3% of the variance in most traits (except the branchial traits for which we only measured a small subset of the fish), none of the other branchial, lateral line, metric, or meristic traits previously mapped to chromosome IV and measured in the Lake Washington fish show an association with the *Eda* haplotype (Table S2).

We also performed fine mapping for geometric morphometric traits in Lake Washington. This analysis revealed that landmarks Y10, X16, and Y18 are associated with the *Eda* haplotype (Fig. S4; Tables 1 and S2). Landmark 10 is the dorsal posterior extent of the operculum, and the X position has previously been mapped to near *Eda* (Albert et al. 2008). Landmarks 18 and 16 mark the anterior and posterior extent of the maxilla, respectively. The Y position of landmark 16 has previously been mapped to chromosome IV (Albert et al. 2008).

### LARGE EFFECT TRAITS MAP TO THE SAME INTRAGENIC REGION OF *Eda*

We find that the phenotypes that map strongly to *Eda* all have the same association pattern with the markers across the 16‐kb haplotype. The strongest associations are with indels LP3621 and Cnv770, which are located in the first intron of *Eda* (Figs. 1 and 6). These data suggest that one or more closely linked mutations in a 1.4‐kb region mediate the effects of the *Eda* haplotype on left plate count, D‐V neuromast patterning, and Mp neuromasts per segment. Notably, the lowest LOD score for all three traits is at the NAKA SNP, consistent with the fact that the NAKA SNP is not sufficient to affect plate count or lateral line phenotypes in the Puget Sound crosses (Figs. 5 and 6).

### NUMBER OF LATERAL PLATES IS LIKELY AFFECTED BY MULTIPLE MUTATIONS WITHIN THE HAPLOTYPE

**Figure 7:**
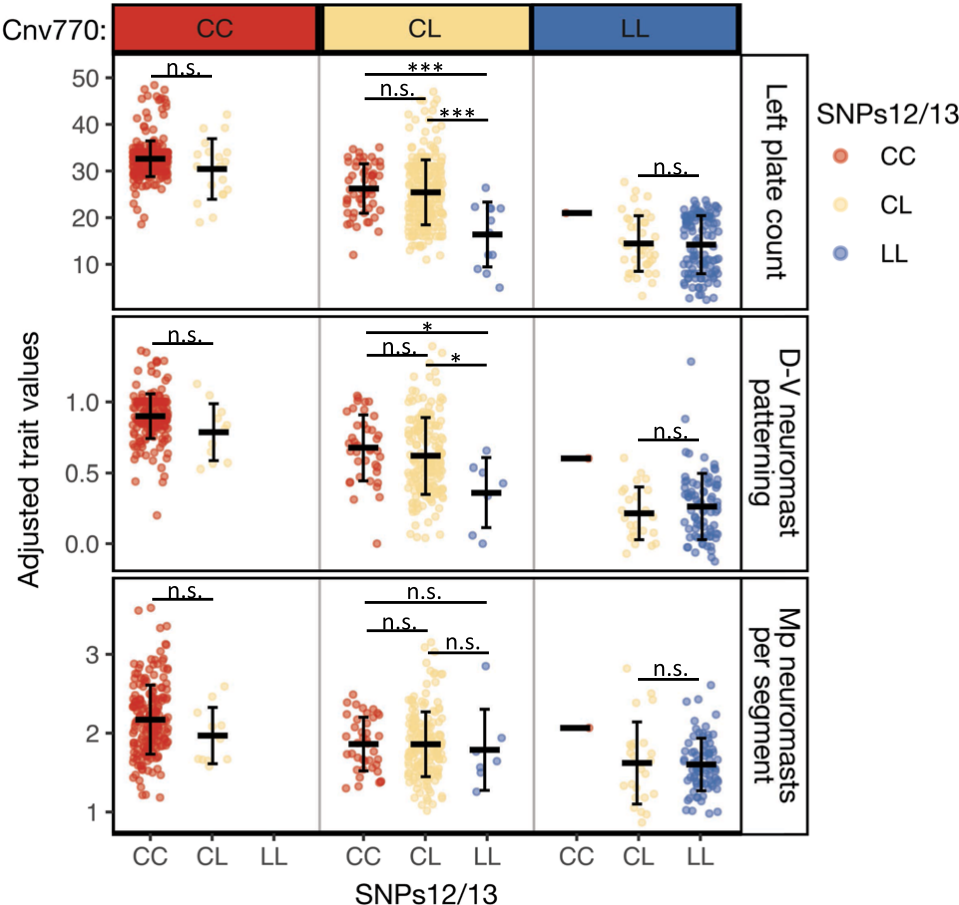
**SNPs12/13 explain additional variation in plate count and D‐V neuromast patterning of Lake Washington stickleback after accounting for Cnv770**. Adjusted left plate count, D‐V neuromast pattern, and Mp neuromasts per segment are plotted for Lake Washington fish by both genotype at Cnv770 (the marker with the highest single LOD score in the association mapping) and SNPs12/13, which controlled additional variation in plate count in the Puget Sound crosses (Fig. 3C). Mean trait value ± SD are depicted by black lines and whiskers. Fish that are heterozygous at both Cnv770 and SNPs12/13 have significantly more plates than fish heterozygous at Cnv770 and homozygous LL at SNPs12/13 (*t*_12_ = 4.4, *P* = 0.00085) and differ in D‐V neuromast patterning (*t*_12_ = 2.7, *P* = 0.03). The trait values plotted are adjusted for sex, standard length, and collection, and therefore vary outside the normal range of values. There was a single fish with a historical recombination event on both chromosomes between Cnv770 and SNPs12/13, resulting in an LL genotype at Cnv770 and a CC genotype at SNPs12/13. Significance levels: ^*^^*^^*^ *P* < 0.001; ^*^^*^ *P* < 0.01; ^*^ *P* < 0.05; *P* > 0.05 (not significant).

There is a second peak in LOD score at SNP7, which has a higher LOD score than both Stn381 and LP13173 in the analysis using the filled genotype file (data not shown). Together with the finding that Puget Sound F1 offspring with the full‐L haplotype have fewer plates than F1 offspring with the short‐L haplotype (Fig. 3C; Table S3), we hypothesized that there may be more than one mutation within the haplotype that affects plate number. To test this idea, we plotted adjusted left plate counts for the Lake Washington fish by genotype at Cnv770 (highest LOD score in the association mapping) and SNPs12/13 (to be consistent with the analysis of the Puget Sound crosses). We found that genotype at SNPs12/13 explains additional variation in adjusted plate number within fish that are heterozygous at Cnv770 (Fig. 7; Table S3). Although the phenotypic values for neuromast pattern and neuromast number trended in the same direction as the pattern described for lateral plates, significant effects of SNPs12/13 were only observed on neuromast pattern (Fig. 7; Table S3).

## Discussion

The goal of this study was to determine whether a single adaptive haplotype within a phenotypic hotspot is responsible for multiple phenotypic changes, or whether the overlap of these phenotypic and genomic divergence signals is due to the low resolution of QTL mapping. Further, we asked whether the pleiotropic effects of the *Eda* gene are due to pleiotropic effects of a single mutation, or due to linked causative mutations within the adaptive haplotype. First, we found that the haplotype is responsible for two traits previously known to be affected by *Eda* (lateral plate count and neuromast patterning), plus the number of neuromasts per body segment. We did not find evidence that the haplotype explains significant variation in additional phenotypes that have previously mapped to chromosome IV. Second, we found evidence for a small 1.4‐kb region with large effects on both neuromast and bony armor traits, as well as additional linked, small effect mutations that affect bony armor, feeding, and shape traits.

### PLEIOTROPIC EFFECTS OF A 1.4‐kb REGION IN THE *Eda* HAPLOTYPE ON LATERAL PLATE AND LATERAL LINE TRAITS

Both the Puget Sound and Lake Washington datasets suggest that variation in lateral bony plate number, dorsal‐ventral patterning, and number of neuromasts per segment in the main posterior lateral line maps to a small genomic region with large effects. These effects are therefore due to the shared freshwater haplotype, rather than population‐specific alleles or gene by environment interactions. The strong similarity of the LOD curves for these traits suggests that the pleiotropic effects of a single or few tightly linked mutations at or between LP3621 and Cnv770 are responsible. This is consistent with the Puget Sound data that also show that these traits map to the short‐L haplotype, which includes LP3621 and Cnv770.

The pleiotropic effects of this region may be mediated either via (1) clustered mutations that alter distinct but closely linked regulatory regions that independently control armor plate formation and neuromast number and patterning; (2) direct interactions between the developing tissues/cell types responsible for the different traits; or (3) via independent responses by the relevant tissues to the same external signal. All three hypotheses are consistent with the presence of multiple causative mutations; however, in the latter two cases, each mutation would need to have pleiotropic effects on both plate and neuromast development. We favor these latter two hypotheses that mutation(s) have pleiotropic effects because plate development and neuromast patterning are tightly correlated in space and time during stickleback development. Armor plates form around neuromasts, and neuromasts display the dorsal‐ventral arrangement only on body segments with plates (Wark and Peichel 2010; Wark et al. 2012; Mills et al. 2014). In support of the second hypothesis, work in zebrafish has shown that neuromasts and dermal bone, such as the opercular bone and scales, interact (Wada et al. 2010; Wada et al. 2014). However, the interaction between lateral scale formation (thought to be homologous to lateral plate formation in stickleback) and patterning or number of neuromasts has not been investigated. In support of the third hypothesis, Wnt signaling is required for neuromast proliferation and scale formation in zebrafish (Wada et al. 2013; Lush and Piotrowski 2014; Aman et al. 2018), as well as for normal plate patterning in sticklebacks (O'Brown et al. 2015). *Eda* is required for refinement of Wnt expression prior to scale formation (Aman et al. 2018), but its role in neuromast patterning or proliferation has not been investigated. Therefore, lateral plate and neuromast traits could be responding independently to the modification of the same external (Wnt) signal caused by a reduction in *Eda* expression in freshwater sticklebacks. Studying neuromast patterning in *Eda* mutants or studying the interactions between scale/plate formation and neuromast patterning in species with natural variation in these traits may be informative for disentangling the latter two hypotheses. Ultimately, determining whether the pleiotropic effects of this region are due to a single or linked mutations will require further resolution of the sequence changes responsible for armor plate and neuromast changes in sticklebacks.

The assumption that individual mutations have pleiotropic effects is central to Fisher's model of adaptation and the prediction that complex organisms pay a cost in the rate of adaptation (Fisher 1930; Orr 2000). Dissecting the phenotypic effects of individual mutations found in pleiotropic regions of the genome is therefore critical to validating our theoretical assumptions. However, few adaptive pleiotropic mutations are known (Sabeti et al. 2007; Kamberov et al. 2013; Nagy et al. 2018; Butelli et al. 2019; Ramaekers et al. 2019), so identifying the particular mutation(s) behind adaptive plate loss and neuromast changes will be an informative endeavor. Previous sequence conservation analysis and allele‐specific expression work suggested that the causative mutation for plate loss is a regulatory mutation that drives higher expression of the marine *Eda* allele in the flank of the fish, but not in other body regions, such as the midline fins (Colosimo et al. 2005; O'Brown et al. 2015). This suggests that the causative mutation is in a tissue‐specific enhancer of *Eda*. A single SNP that is shared between North American and Japanese freshwater, low‐plated stickleback populations, the NAKA SNP, is within a flank enhancer and displayed reduced responsiveness to Wnt signaling compared to the marine enhancer (O'Brown et al. 2015). The frequency of the L allele at the NAKA SNP in the Puget Sound marine population sampled in 2015 was 9.4%. However, our crosses between heterozygous carriers of the NAKA SNP demonstrated that the NAKA SNP is not sufficient to cause phenotypic changes in plate number or neuromast patterning (Fig. 5). Reinforcing this finding, the NAKA SNP had the lowest LOD score within the haplotype for plate count and neuromast patterning in our association mapping study within Lake Washington (Fig. 6). Recent population sampling in Japan confirms that the freshwater allele of the NAKA SNP is found in all sampled freshwater populations with the low‐plated phenotype, but also occurs in the closely related marine species *G. nipponicus*, which is completely plated (Yamasaki et al. 2019). In addition, the freshwater allele of the NAKA SNP was found in multiple percomorph fish taxa that have scales, including tilapia and platyfish (Fig. S5), suggesting that the marine allele of the SNP is derived. Thus, the functional consequences of the NAKA SNP remain a mystery.

In addition to ruling out the NAKA SNP as the causative mutation for plate loss and neuromast number and patterning, we were able to narrow the putative causative region to intron 1 of *Eda*, which is consistent with a causative mutation in an enhancer of *Eda*. The LOD curves from the association mapping study suggest that the causative mutation(s) are conservatively between Stn382 and SNP5, corresponding to bases 12,802,847‐12,808,303 in the gasAcu1 genome assembly. Additionally, alignment of all low‐plated Lake Washington fish has identified a smaller putative causative region that includes approximately 19 polymorphisms that differentiate the marine and freshwater *Eda* haplotypes and spans a 1401‐bp region (Fig. S6). This region includes two indels from this study: LP3621 and Cnv770, which encode a 16 bp and a 107 bp deletion in the freshwater and marine haplotypes, respectively (Lowe et al. 2018). Genetic manipulations are now underway to test whether this intronic region contains one or more enhancers active near developing lateral plates or neuromasts, and if so, to test which SNPs or indels may alter the activity of these enhancers and drive the phenotypic differences in plate number, neuromast number, and neuromast patterning.

### LINKED MUTATIONS IN THE *Eda* HAPLOTYPE HAVE SMALLER EFFECTS ON MULTIPLE TRAITS

In addition to identifying a small 1.4‐kb region within intron 1 that causes phenotypic changes in three traits, we found evidence of linked mutations with effects on the same phenotypes as well as additional phenotypes. Data from the Puget Sound crosses suggest that there is a mutation in the downstream portion (∼3000 bp) of the haplotype that has a small effect on gill raker length (Fig. S7). We did not recover this correlation in the Lake Washington mapping population, either due to absence of that mutation, epistatic interactions, or phenotypic plasticity in the wild‐caught fish (Day et al. 1994). However, we did find associations in the Lake Washington population between three geometric morphometric landmarks and the *Eda* haplotype. Two of these landmark positions (Y10 and Y18) had very similar LOD curves (Fig. S4), suggestive of another mutation with pleiotropic effects. Geometric morphometric analysis of the Puget Sound crosses would tell us if these associations hold for the freshwater haplotype in different genetic backgrounds and populations. Because the PVE of these linked mutations is much lower than the PVE from previous QTL mapping studies (Albert et al. 2008), we do not think we have mapped the previously identified QTL for these traits.

Nonetheless, it is possible that the *Eda* haplotype may contain additional mutations of very small effect. Despite having high power to detect mutations that explain 5% or less of the variation for most traits in our study (Table S2), we cannot rule out the possibility that there are mutations with weak effect within the region. In fact, 44 of 91 traits (48%) measured in the Lake Washington population had *P*‐values less than 0.05 but did not meet our significance correction for multiple comparisons (Table S2). The high proportion of traits in this category is consistent with the idea that most causative mutations have weak and nearly undetectable effects on phenotype (Rockman 2012).

We also found evidence in both the Puget Sound crosses and the Lake Washington mapping population that additional linked mutation(s) have effects on plate count and neuromast patterning. Together with linked mutations affecting morphometric landmarks, this could explain why the 16‐kb full‐L version of the freshwater haplotype is commonly favored and fixed in most low‐plated populations. The size of the 16‐kb minimal shared freshwater haplotype could be due to either selection in freshwater for the full‐L haplotype or due to lack of recombination events that make the haplotype smaller. We found that short‐L haplotypes were nearly as abundant as full‐L freshwater haplotypes in the nearshore Puget Sound marine population sampled in 2016, suggesting that recombination has not limited the availability of shorter haplotypes in anadromous fish (and consequently in freshwater populations). Furthermore, in Lake Washington, where anthropogenic activity has led to a recent shift in selection toward favoring completely plated individuals and therefore the marine genotype at *Eda* (Kitano et al. 2008), we find that 22% of individuals have a recombination event within the 16‐kb haplotype. These data suggest that recombination is not limiting in this genomic region. Therefore, selection may be preserving the full 16‐kb freshwater haplotype in typical freshwater environments due to the multiple mutations with phenotypic effects.

### SELECTION ON THE *Eda* HAPLOTYPE IN FRESHWATER

Because changes in neuromast number, patterning, and plate count all map to the same small genomic interval, we cannot conclude which trait(s) provide a fitness advantage in freshwater. Neuromast number in the posterior lateral line is variable across freshwater populations and can exceed neuromast number in marine populations (Wark and Peichel 2010; Jiang et al. 2016). This suggests that selection on this trait is not driven by shared conditions across freshwater environments and is likely not the main target of selection within the *Eda* haplotype. The fitness effects of the dorsal‐ventral patterning of neuromasts are unknown, although it may play a role in schooling behavior (Greenwood et al. 2016). In contrast, much attention has been given to the possible role of plate loss in adaptation to freshwater. Many bony elements are reduced in freshwater, suggesting that an overall reduction in bone may be advantageous (Giles 1983; Bell et al. 1993; Bell and Foster 1994; Myhre and Klepaker 2009). In addition, plates play a known functional role in predation survival, which confirms their visibility to selection in certain environments (Reimchen 1992). Although there are known ecological correlates with plate reduction in freshwater environments, such as ion concentration, distance from ocean, and co‐occurrence with predators, the selection pressure acting to reduce number of lateral plates is unknown (Bell et al. 1993; Bourgeois et al. 1994; Gelmond et al. 2009). It is also possible that there are pleiotropic effects of this mutation on other, as yet unmeasured phenotypes, which are the direct targets of selection (Barrett et al. 2008, 2009; Rennison et al. 2015).

More broadly our results suggest that the phenotypic hotspot on chromosome IV (Peichel and Marques 2017) is not explained solely by the *Eda* haplotype. A few traits in previous QTL mapping studies had their highest association within the *Eda* haplotype (Stn382), including ceratobranchial length and dorsal pharyngeal tooth number (Erickson et al. 2014; Miller et al. 2014), and induced coding region mutations in the stickleback *Eda* gene eliminate armor plates and significantly reduce pharyngeal teeth (Wucherpfennig et al. 2019). However, even with our reduced power to detect QTL of small effect for these branchial traits, there was no evidence for suggestive associations between these traits and genotype at the *Eda* haplotype in our study (Table S2). Although we cannot rule out a mutation within the freshwater haplotype that is specific to the populations used in the previous QTL crosses (Pacific Ocean marine from Japan and Paxton benthic freshwater from Canada), our results suggest that additional linked mutations outside, but near, the *Eda* haplotype contribute to these other phenotypic traits that map to chromosome IV. For example, there is a dorsal spine QTL approximately 1 Mb downstream from *Eda* corresponding to the gene *Msx2a* (Howes et al. 2017). Linkage of multiple causative mutations within and near *Eda* is consistent with the prediction that linked adaptive mutations are favored in the presence of gene flow (Charlesworth and Charlesworth 1979; Kirkpatrick and Barton 2006; Yeaman and Whitlock 2011).

## Conclusions

Pleiotropic mutations have been proposed as a limitation to adaptation due to their potential deleterious effects on additional traits, yet pleiotropic loci appear to be common. We have measured the phenotypic effects of an adaptive haplotype within a phenotypic hotspot in two separate populations and found that it contains a small genomic region controlling three traits—lateral plate count, neuromast number, and neuromast pattern—and additional mutations with small effects on lateral plate count and body shape. We propose that that the multiple phenotypes controlled by this small genomic region facilitate rapid adaptation and that selection favors the entire haplotype in freshwater due to the linkage of multiple mutations.

## CONFLICT OF INTEREST

The authors declare no conflict of interest.

## AUTHOR CONTRIBUTIONS

SLA and CLP conceived and designed the study. SLA, LRB, and ADM collected and analyzed the data. SLA and CLP wrote the manuscript with contributions from LRB and ADM.

## DATA ARCHIVING

All of the datasets and the R code for analyses are deposited on Dryad: https://doi.org/10.5061/dryad.05qfttf0j.

Associate Editor: J. Mank

## Supporting information


**Figure S1**. Twelve neuromast lateral lines were phenotyped using either DASPEI or alkaline phosphatase staining to identify and count neuromasts.
**Figure S2**. Landmark positions and descriptions used for geometric morphometrics.
**Figure S3**. Visual genotypes of Lake Washington fish reveal historical recombination events and frequency of NAKA SNP.
**Figure S4**. Association mapping of geometric morphometric traits in Lake Washington stickleback reveals three traits that are associated with the *Eda* haplotype.
**Figure S5**. NAKA SNP near *Eda* is conserved across most percomorph species and likely lost in marine stickleback.
**Figure S6**. Recombination breakpoints in low‐plated Lake Washington fish narrows the region containing the causative mutation to 1400 base pairs.
**Figure S7**. Gill raker length maps to SNPs12/13 in Puget Sound.
**Table S1**. Summary of genotyping assays used in this study.
**Table S2**. Summary of mapping results performed in this study.
**Table S3**. Estimated effects of Cnv770 and SNPs12/13 on traits that map to the haplotype.

Supplementary Material

## References

[evl3175-bib-0001] Albert, A. Y. K. , S. Sawaya , T. H. Vines , A. K. Knecht , C. T. Miller , B. R. Summers , et al. 2008 The genetics of adaptive shape shift in stickleback: pleiotropy and effect size. Evolution 62:76–85. 18005154 10.1111/j.1558-5646.2007.00259.x

[evl3175-bib-0002] Albertson, R. C. , J. T. Streelman , and T. D. Kocher . 2003 Directional selection has shaped the oral jaws of Lake Malawi cichlid fishes. Proc. Natl. Acad. Sci. USA 100:5252–5257. 12704237 10.1073/pnas.0930235100 PMC154331

[evl3175-bib-0003] Aman, A. J. , A. N. Fulbright , and D. M. Parichy . 2018 Wnt/β‐catenin regulates an ancient signaling network during zebrafish scale development. eLife 7:e37001. 30014845 10.7554/eLife.37001 PMC6072442

[evl3175-bib-0004] Barrett, R. D. H. , and H. E. Hoekstra . 2011 Molecular spandrels: tests of adaptation at the genetic level. Nat. Rev. Genet. 12:767–780. 22005986 10.1038/nrg3015

[evl3175-bib-0005] Barrett, R. D. H. , S. M. Rogers , and D. Schluter . 2008 Natural selection on a major armor gene in threespine stickleback. Science 322:255–257. 18755942 10.1126/science.1159978

[evl3175-bib-0006] Barrett, R. D. H. , S. M. Rogers , and D. Schluter. 2009 Environment specific pleiotropy facilitates divergence at the *Ectodysplasin* locus in threespine stickleback. Evolution 63:2831–2837. 19545262 10.1111/j.1558-5646.2009.00762.x

[evl3175-bib-0007] Bassham, S. , J. Catchen , E. Lescak , F. A. von Hippel , and W. A. Cresko . 2018 Repeated selection of alternatively adapted haplotypes creates sweeping genomic remodeling in stickleback. Genetics 209:921–939. 29794240 10.1534/genetics.117.300610 PMC6028257

[evl3175-bib-0008] Bell, M. A. , and S. A. Foster . 1994 The evolutionary biology of the threespine stickleback. Oxford Univ. Press, New York.

[evl3175-bib-0009] Bell, M. A. , G. Orti , J. A. Walker , and J. P. Koenings . 1993 Evolution of pelvic reduction in threespine stickleback fish: a test of competing hypotheses. Evolution 47:906–914. 28567888 10.1111/j.1558-5646.1993.tb01243.x

[evl3175-bib-0010] Bourgeois, J. F. , D. M. Blouw , J. P. Koenings , and M. A. Bell . 1994 Multivariate analysis of geographic covariance between phenotypes and environments in the threespine stickleback, *Gasterosteus aculeatus*, from the Cook Inlet area, Alaska. Can. J. Zool. 72:1497–1509.

[evl3175-bib-0011] Boyle, E. A. , Y. I. Li , and J. K. Pritchard . 2017 An expanded view of complex traits: from polygenic to omnigenic. Cell 169:1177–1186. 28622505 10.1016/j.cell.2017.05.038 PMC5536862

[evl3175-bib-0012] Bratteler, M. , C. Lexer , and A. Widmer . 2006 Genetic architecture of traits associated with serpentine adaptation of *Silene vulgaris* . J. Evol. Biol. 19:1149–1156. 16780515 10.1111/j.1420-9101.2006.01090.x

[evl3175-bib-0013] Broman, K. W. , and S. Sen . 2009 A guide to QTL mapping with R/qtl. Springer‐Verlag, New York.

[evl3175-bib-0014] Butelli, E. , C. Licciardello , C. Ramadugu , M. Durand‐Hulak , A. Celant , G. Reforgiato Recupero , et al. 2019 *Noemi* controls production of flavonoid pigments and fruit acidity and illustrates the domestication routes of modern citrus varieties. Curr. Biol. 29:158–164. 30581020 10.1016/j.cub.2018.11.040

[evl3175-bib-0015] Carbone, M. A. , K. W. Jordan , R. F. Lyman , S. T. Harbison , J. Leips , T. J. Morgan , et al. 2006 Phenotypic variation and natural selection at *Catsup*, a pleiotropic quantitative trait gene in *Drosophila* . Curr. Biol. 16:912–919. 16682353 10.1016/j.cub.2006.03.051 PMC10766118

[evl3175-bib-0016] Carroll, S. B. 2008 Evo‐devo and an expanding evolutionary synthesis: a genetic theory of morphological evolution. Cell 134:25–36. 18614008 10.1016/j.cell.2008.06.030

[evl3175-bib-0017] Chan, Y. F. , M. E. Marks , F. C. Jones , G. Villarreal Jr. , M. D. Shapiro , S. D. Brady , et al. 2010 Adaptive evolution of pelvic reduction in sticklebacks by recurrent deletion of a *Pitx1* enhancer. Science 327:302–305. 20007865 10.1126/science.1182213 PMC3109066

[evl3175-bib-0018] Charlesworth, D. , and B. Charlesworth . 1979 Selection on recombination in clines. Genetics 91:581–589. 17248899 10.1093/genetics/91.3.581 PMC1216851

[evl3175-bib-0019] Chesmore, K. , J. Bartlett , and S. M. Williams . 2018 The ubiquity of pleiotropy in human disease. Hum. Genet. 137:39–44. 29164333 10.1007/s00439-017-1854-z

[evl3175-bib-0020] Cocker, J. M. , J. Wright , J. Li , D. Swarbreck , S. Dyer , M. Caccamo , et al. 2018 *Primula vulgaris* (primrose) genome assembly, annotation and gene expression, with comparative genomics on the heterostyly supergene. Sci. Rep. 8:17942. 30560928 10.1038/s41598-018-36304-4 PMC6299000

[evl3175-bib-0021] Colosimo, P. F. , K. E. Hosemann , S. Balabhadra , G. Villarreal Jr. , M. Dickson , J. Grimwood , et al. 2005 Widespread parallel evolution in sticklebacks by repeated fixation of *Ectodysplasin* alleles. Science 307:1928–1933. 15790847 10.1126/science.1107239

[evl3175-bib-0022] Colosimo, P. F. , C. L. Peichel , K. Nereng , B. K. Blackman , M. D. Shapiro , D. Schluter , et al. 2004 The genetic architecture of parallel armor plate reduction in threespine sticklebacks. PLoS Biol. 2:635–641. 10.1371/journal.pbio.0020109 PMC385219 15069472

[evl3175-bib-0023] Darwin, C. 1859 On the origin of species by means of natural selection. John Murray, Lond.

[evl3175-bib-0024] Day, T. , J. Pritchard , and D. Schluter . 1994 Ecology and genetics of phenotypic plasticity: a comparison of two sticklebacks. Evolution 48:1723–1734. 28568405 10.1111/j.1558-5646.1994.tb02208.x

[evl3175-bib-0025] Dong, Y. , J. Liu , P.‐W. Li , C.‐Q. Li , T.‐F. Lü , X. Yang , et al. 2018 Evolution of Darwin's *peloric* Gloxinia (*Sinningia speciosa*) is caused by a null mutation in a pleiotropic TCP gene. Mol. Biol. Evol. 35:1901–1915. 29718509 10.1093/molbev/msy090 PMC6063280

[evl3175-bib-0026] Ellis, N. A. , and C. T. Miller . 2016 Dissection and flat‐mounting of the threespine stickleback branchial skeleton. J. Vis. Exp. 10.3791/54056. PMC4942055 27213248

[evl3175-bib-0027] Erickson, P. A. , A. M. Glazer , P. A. Cleves , A. S. Smith , and C. T. Miller . 2014 Two developmentally temporal quantitative trait loci underlie convergent evolution of increased branchial bone length in sticklebacks. Proc. R. Soc. Lond. B 281:20140822. 10.1098/rspb.2014.0822 PMC4083797 24966315

[evl3175-bib-0028] Erickson, P. A. , J. Baek , J. C. Hart , P. A. Cleves , and C. T. Miller . 2018 Genetic dissection of a supergene implicates *Tfap2a* in craniofacial evolution of threespine sticklebacks. Genetics 209:591–605. 29593029 10.1534/genetics.118.300760 PMC5972429

[evl3175-bib-0029] Fisher, R. A. 1930 The genetical theory of natural selection. Clarendon Press, Oxford, U.K.

[evl3175-bib-0030] Fishman, L. , A. Stathos , P. M. Beardsley , C. F. Williams , and J. P. Hill . 2013 Chromosomal rearrangements and the genetics of reproductive barriers in *Mimulus* (monkeyflowers). Evolution 67:2547–2560. 24033166 10.1111/evo.12154

[evl3175-bib-0031] Frankel, N. , D. F. Erezyilmaz , A. P. McGregor , S. Wang , F. Payre , and D. L. Stern . 2011 Morphological evolution caused by many subtle‐effect substitutions in regulatory DNA. Nature 474:598–603. 21720363 10.1038/nature10200 PMC3170772

[evl3175-bib-0032] Friedman, J. , A. D. Twyford , J. H. Willis , and B. K. Blackman . 2015 The extent and genetic basis of phenotypic divergence in life history traits in *Mimulus guttatus* . Mol. Ecol. 24:111–122. 25403267 10.1111/mec.13004 PMC4657477

[evl3175-bib-0033] Gelmond, O. , F. A. Von Hippel , and M. S. Christy . 2009 Rapid ecological speciation in three‐spined stickleback *Gasterosteus aculeatus* from Middleton Island, Alaska: the roles of selection and geographic isolation. J. Fish Biol. 75:2037–2051. 20738670 10.1111/j.1095-8649.2009.02417.x

[evl3175-bib-0034] Giles, N. 1983 The possible role of environmental calcium levels during the evolution of phenotypic diversity in Outer Hebridean populations of the three‐spined stickleback, *Gasterosteus aculeatus* . J. Zool. 199:535–544.

[evl3175-bib-0035] Greenwood, A. K. , A. R. Wark , K. Yoshida , and C. L. Peichel . 2013 Genetic and neural modularity underlie the evolution of schooling behavior in threespine sticklebacks. Curr. Biol. 23:1884–1888. 24035541 10.1016/j.cub.2013.07.058 PMC3828509

[evl3175-bib-0036] Greenwood, A. K. , M. G. Mills , A. R. Wark , S. L. Archambeault , and C. L. Peichel . 2016 Evolution of schooling behavior in threespine sticklebacks is shaped by the *Eda* gene. Genetics 203:677–681. 27052567 10.1534/genetics.116.188342 PMC4896186

[evl3175-bib-0037] Hagen, D. W. , and L. G. Gilbertson . 1972 Geographic variation and environmental selection in *Gasterosteus aculeatus* L. in the Pacific Northwest, America. Evolution 26:32–51. 28555771 10.1111/j.1558-5646.1972.tb00172.x

[evl3175-bib-0038] Hall, M. C. , C. J. Basten , and J. H. Willis . 2006 Pleiotropic quantitative trait loci contribute to population divergence in traits associated with life‐history variation in *Mimulus guttatus* . Genetics 172:1829–1844. 16361232 10.1534/genetics.105.051227 PMC1456280

[evl3175-bib-0039] Harris, M. P. , N. Rohner , H. Schwarz , S. Perathoner , P. Konstantinidis , and C. Nusslein‐Volhard . 2008 Zebrafish *eda* and *edar* mutants reveal conserved and ancestral roles of ectodysplasin signaling in vertebrates. PLoS Genet. 4:e1000206. 18833299 10.1371/journal.pgen.1000206 PMC2542418

[evl3175-bib-0040] Hawthorne, D. J. , and S. Via . 2001 Genetic linkage of ecological specialization and reproductive isolation in pea aphids. Nature 412:904–907. 11528477 10.1038/35091062

[evl3175-bib-0041] Hendry, A. P. , C. L. Peichel , B. Matthews , J. W. Boughman , and P. Nosil . 2013 Stickleback research: the now and the next. Evol. Ecol. Res. 15:111–141.

[evl3175-bib-0042] Hermann, K. , U. Klahre , M. Moser , H. Sheehan , T. Mandel , and C. Kuhlemeier . 2013 Tight genetic linkage of prezygotic barrier loci creates a multifunctional speciation island in *Petunia* . Curr. Biol. 23:873–877. 23602480 10.1016/j.cub.2013.03.069

[evl3175-bib-0043] Hoekstra, H. E. , and J. A. Coyne . 2007 The locus of evolution: evo devo and the genetics of adaptation. Evolution 61:995–1016. 17492956 10.1111/j.1558-5646.2007.00105.x

[evl3175-bib-0044] Hoffmann, A. A. , and L. H. Rieseberg . 2008 Revisiting the impact of inversions in evolution: from population genetic markers to drivers of adaptive shifts and speciation? Ann. Rev. Ecol. Evol. Syst. 39:21–42. 20419035 10.1146/annurev.ecolsys.39.110707.173532 PMC2858385

[evl3175-bib-0045] Hohenlohe, P. A. , S. Bassham , P. D. Etter , N. Stiffler , E. A. Johnson , and W. A. Cresko . 2010 Population genomics of parallel adaptation in threespine stickleback using sequenced RAD tags. PLoS Genet. 6:e1000862. 20195501 10.1371/journal.pgen.1000862 PMC2829049

[evl3175-bib-0046] Howes, T. R. , B. R. Summers , and D. M. Kingsley . 2017 Dorsal spine evolution in threespine sticklebacks via a splicing change in *MSX2A* . BMC Biol. 15:115. 29212540 10.1186/s12915-017-0456-5 PMC5719529

[evl3175-bib-0047] Jiang, Y. , C. L. Peichel , F. Ling , and D. I. Bolnick . 2016 Sensory trait variation contributes to biased dispersal of threespine stickleback in flowing water. J. Evol. Biol. 30:681–695. 10.1111/jeb.13035 28029723

[evl3175-bib-0048] Jones, F. C. , M. G. Grabherr , Y. F. Chan , P. Russell , E. Mauceli , J. Johnson , et al. 2012 The genomic basis of adaptive evolution in threespine sticklebacks. Nature 484:55–61. 22481358 10.1038/nature10944 PMC3322419

[evl3175-bib-0049] Joron, M. , L. Frezal , R. T. Jones , N. L. Chamberlain , S. F. Lee , C. R. Haag , et al. 2011 Chromosomal rearrangements maintain a polymorphic supergene controlling butterfly mimicry. Nature 477:203–206. 21841803 10.1038/nature10341 PMC3717454

[evl3175-bib-0050] Kamberov, Y G. , S. Wang , J. Tan , P. Gerbault , A. Wark , L. Tan , et al. 2013 Modeling recent human evolution in mice by expression of a selected EDAR variant. Cell 152:691–702. 23415220 10.1016/j.cell.2013.01.016 PMC3575602

[evl3175-bib-0051] Kearse, M. , R. Moir , A. Wilson , S. Stones‐Havas , M. Cheung , S. Sturrock , et al. 2012 Geneious Basic: an integrated and extendable desktop software platform for the organization and analysis of sequence data. Bioinformatics 28:1647–1649. 22543367 10.1093/bioinformatics/bts199 PMC3371832

[evl3175-bib-0052] Kingsley, D. M. , and C. L. Peichel . 2007 The molecular genetics of evolutionary change in sticklebacks Pp. 41–81 *in* Ostlund‐Nilsson S., Mayer I., and Huntingford F. A., eds. Biology of the three‐spined stickleback. CRC Press, Boca Raton, FL.

[evl3175-bib-0053] Kirkpatrick, M. , and N. Barton . 2006 Chromosome inversions, local adaptation and speciation. Genetics 173:419–434. 16204214 10.1534/genetics.105.047985 PMC1461441

[evl3175-bib-0054] Kitano, J. , D. I. Bolnick , D. A. Beauchamp , M. M. Mazur , S. Mori , T. Nakano , et al. 2008 Reverse evolution of armor plates in the threespine stickleback. Curr. Biol. 18:769–774. 18485710 10.1016/j.cub.2008.04.027

[evl3175-bib-0055] Kunte, K. , W. Zhang , A. Tenger‐Trolander , D. H. Palmer , A. Martin , R. D. Reed , et al. 2014 *doublesex* is a mimicry supergene. Nature 507:229–232. 24598547 10.1038/nature13112

[evl3175-bib-0056] Küpper, C. , M. Stocks , J. E. Risse , N. dos Remedios , L. L. Farrell , S. B. McRae , et al. 2016 A supergene determines highly divergent male reproductive morphs in the ruff. Nat. Genet. 48:79–83. 26569125 10.1038/ng.3443 PMC5218575

[evl3175-bib-0057] Lamichhaney, S. , G. Fan , F. Widemo , U. Gunnarsson , D. S. Thalmann , M. P. Hoeppner , et al. 2016 Structural genomic changes underlie alternative reproductive strategies in the ruff (*Philomachus pugnax*). Nat. Genet. 48:84–88. 26569123 10.1038/ng.3430

[evl3175-bib-0058] Lee, C. ‐ R. , B. Wang , J. P. Mojica , T. Mandáková , K. V. S. K. Prasad , J. L. Goicoechea , et al. 2017 Young inversion with multiple linked QTLs under selection in a hybrid zone. Nat. Ecol. Evol. 1:119. 28812690 10.1038/s41559-017-0119 PMC5607633

[evl3175-bib-0059] Lewis, J. J. , R. C. Geltman , P. C. Pollak , K. E. Rondem , S. M. Van Belleghem , M. J. Hubisz , et al. 2019 Parallel evolution of ancient, pleiotropic enhancers underlies butterfly wing pattern mimicry. Proc. Natl. Acad. Sci. USA 116:24174–24183. 31712408 10.1073/pnas.1907068116 PMC6883815

[evl3175-bib-0060] Linnen, C. R. , Y. P. Poh , B. K. Peterson , R. D. Barrett , J. G. Larson , J. D. Jensen , et al. 2013 Adaptive evolution of multiple traits through multiple mutations at a single gene. Science 339:1312–1316. 23493712 10.1126/science.1233213 PMC3836219

[evl3175-bib-0061] Lowe, C. B. , N. Sanchez‐Luege , T. R. Howes , S. D. Brady , R. R. Richardson , F. C. Jones , et al. 2018 Detecting differential copy number variation between groups of samples. Genome Res. 28:256–265. 29229672 10.1101/gr.206938.116 PMC5793789

[evl3175-bib-0062] Lowry, D. B. , and J. H. Willis . 2010 A widespread chromosomal inversion polymorphism contributes to a major life‐history transition, local adaptation, and reproductive isolation. PLoS Biol. 8:e1000500. 20927411 10.1371/journal.pbio.1000500 PMC2946948

[evl3175-bib-0063] Lush, M. E. , and T. Piotrowski . 2014 ErbB expressing Schwann cells control lateral line progenitor cells via non‐cell‐autonomous regulation of Wnt/β‐catenin. eLife 3:e01832. 24642408 10.7554/eLife.01832 PMC3957165

[evl3175-bib-0064] McKay, J. K. , J. H. Richards , and T. Mitchell‐Olds . 2003 Genetics of drought adaptation in *Arabidopsis thaliana*: I. Pleiotropy contributes to genetic correlations among ecological traits. Mol. Ecol. 12:1137–1151. 12694278 10.1046/j.1365-294x.2003.01833.x

[evl3175-bib-0065] Meeker, N. D. , S. A. Hutchinson , L. Ho , and N. S. Trede . 2007 Method for isolation of PCR‐ready genomic DNA from zebrafish tissues. BioTechniques 43:610–614. 18072590 10.2144/000112619

[evl3175-bib-0066] Miller, C. T. , A. M. Glazer , B. R. Summers , B. K. Blackman , A. R. Norman , M. D. Shapiro , et al. 2014 Modular skeletal evolution in sticklebacks is controlled by additive and clustered quantitative trait Loci. Genetics 197:405–420. 24652999 10.1534/genetics.114.162420 PMC4012497

[evl3175-bib-0067] Mills, M. G. , A. K. Greenwood , and C. L. Peichel . 2014 Pleiotropic effects of a single gene on skeletal development and sensory system patterning in sticklebacks. EvoDevo 5:5. 24499504 10.1186/2041-9139-5-5 PMC3976036

[evl3175-bib-0068] Myhre, F. , and T. Klepaker . 2009 Body armour and lateral‐plate reduction in freshwater three‐spined stickleback *Gasterosteus aculeatus*: adaptations to a different buoyancy regime? J. Fish Biol. 75:2062–2074. 20738672 10.1111/j.1095-8649.2009.02404.x

[evl3175-bib-0069] Nagy, O. , I. Nuez , R. Savisaar , A. E. Peluffo , A. Yassin , M. Lang , et al. 2018 Correlated evolution of two copulatory organs via a single *cis*‐regulatory nucleotide change. Curr. Biol. 28:3450–3457. 30344115 10.1016/j.cub.2018.08.047 PMC7385753

[evl3175-bib-0070] Nelson, T. C. , and W. A. Cresko . 2018 Ancient genomic variation underlies repeated ecological adaptation in young stickleback populations. Evol. Lett. 2:9–21. 30283661 10.1002/evl3.37 PMC6121857

[evl3175-bib-0071] O'Brown, N. M. , B. R. Summers , F. C. Jones , S. D. Brady , and D. M. Kingsley . 2015 A recurrent regulatory change underlying altered expression and Wnt response of the stickleback armor plates gene *EDA* . eLife 4:e05290. 25629660 10.7554/eLife.05290 PMC4384742

[evl3175-bib-0072] Orr, H. A. 2000 Adaptation and the cost of complexity. Evolution 54:13–20. 10937178 10.1111/j.0014-3820.2000.tb00002.x

[evl3175-bib-0073] Ortiz‐Barrientos, D. , J. Engelstädter , and L. H. Rieseberg . 2016 Recombination rate evolution and the origin of species. Trends Ecol. Evol. 31:226–236. 26831635 10.1016/j.tree.2015.12.016

[evl3175-bib-0074] Otto, S. P. 2004 Two steps forward, one step back: the pleiotropic effects of favoured alleles. Proc. R. Soc. Lond. B 271:705–714. 10.1098/rspb.2003.2635 PMC1691650 15209104

[evl3175-bib-0075] Parnell, N. F. , C. D. Hulsey , and J. T. Streelman . 2012 The genetic basis of a complex functional system. Evolution 66:3352–3366. 23106702 10.1111/j.1558-5646.2012.01688.x PMC3490443

[evl3175-bib-0076] Peichel, C. L. , and D. A. Marques 2017 The genetic and molecular architecture of phenotypic diversity in sticklebacks. Phil. Trans. R. Soc. Lond. B 372:20150486. 27994127 10.1098/rstb.2015.0486 PMC5182418

[evl3175-bib-0077] Peichel, C. L. , K. S. Nereng , K. A. Ohgi , B. L. E. Cole , P. F. Colosimo , C. A. Buerkle , et al. 2001 The genetic architecture of divergence between threespine stickleback species. Nature 414:901–905. 11780061 10.1038/414901a

[evl3175-bib-0078] Peichel, C. L. , J. A. Ross , C. K. Matson , M. Dickson , J. Grimwood , J. Schmutz , et al. 2004 The master sex‐determination locus in threespine sticklebacks is on a nascent Y chromosome. Curr. Biol. 14:1416–1424. 15324658 10.1016/j.cub.2004.08.030

[evl3175-bib-0079] Protas, M. , I. Tabansky , M. Conrad , J. B. Gross , O. Vidal , C. J. Tabin , et al. 2008 Multi‐trait evolution in a cave fish, *Astyanax mexicanus* . Evol. Dev. 10:196–209. 18315813 10.1111/j.1525-142X.2008.00227.x

[evl3175-bib-0080] Ramaekers, A. , A. Claeys , M. Kapun , E. Mouchel‐Vielh , D. Potier , S. Weinberger , et al. 2019 Altering the temporal regulation of one transcription factor drives evolutionary trade‐offs between head sensory organs. Dev. Cell 50:1–13. 31447264 10.1016/j.devcel.2019.07.027

[evl3175-bib-0081] Rebeiz, M. , J. E. Pool , V. A. Kassner , C. F. Aquadro , and S. B. Carroll . 2009 Stepwise modification of a modular enhancer underlies adaptation in a *Drosophila* population. Science 326:1663–1667. 20019281 10.1126/science.1178357 PMC3363996

[evl3175-bib-0082] Reimchen, T. E. 1992 Injuries on stickleback from attacks by a toothed predator (*Oncorhynchus*) and implications for the evolution of lateral plates. Evolution 46:1224–1230. 28564400 10.1111/j.1558-5646.1992.tb00631.x

[evl3175-bib-0083] Rennison, D. J. , K. Heilbron , R. D. H. Barrett , and D. Schluter . 2015 Discriminating selection on lateral plate phenotype and its underlying gene, *Ectodysplasin*, in threespine stickleback. Am. Nat. 185:150–156. 25560560 10.1086/679280

[evl3175-bib-0084] Rockman, M. V. 2012 The QTN program and the alleles that matter for evolution: all that's gold does not glitter. Evolution 66:1–17. 22220860 10.1111/j.1558-5646.2011.01486.x PMC3386609

[evl3175-bib-0085] Sabarís, G. , I. Laiker , E. Preger‐Ben Noon , and N. Frankel . 2019 Actors with multiple roles: pleiotropic enhancers and the paradigm of enhancer modularity. Trends Genet. 35:423–433. 31005339 10.1016/j.tig.2019.03.006

[evl3175-bib-0086] Sabeti, P. C. , P. Varilly , B. Fry , J. Lohmueller , E. Hostetter , C. Cotsapas , et al. 2007 Genome‐wide detection and characterization of positive selection in human populations. Nature 449:913–918. 17943131 10.1038/nature06250 PMC2687721

[evl3175-bib-0087] Scarcelli, N. , J. M. Cheverud , B. A. Schaal , and P. X. Kover . 2007 Antagonistic pleiotropic effects reduce the potential adaptive value of the FRIGIDA locus. Proc. Natl. Acad. Sci. USA 104:16986–16991. 17940010 10.1073/pnas.0708209104 PMC2040464

[evl3175-bib-0088] Schwander, T. , R. Libbrecht , and L. Keller . 2014 Supergenes and complex phenotypes. Curr. Biol. 24:R288–R294. 24698381 10.1016/j.cub.2014.01.056

[evl3175-bib-0089] Sen, Ś. , J. M. Satagopan , K. W. Broman , and G. A. Churchill . 2007 R/qtlDesign: inbred line cross experimental design. Mamm. Genome 18:87–93. 17347894 10.1007/s00335-006-0090-y PMC2366108

[evl3175-bib-0090] Srivastava, A. K. , J. Pispa , A. J. Hartung , Y. Du , S. Ezer , T. Jenks , et al. 1997 The Tabby phenotype is caused by mutation in a mouse homologue of the *EDA* gene that reveals novel mouse and human exons and encodes a protein (ectodysplasin‐A) with collagenous domains. Proc. Natl. Acad. Sci. USA 94:13069–13074. 9371801 10.1073/pnas.94.24.13069 PMC24264

[evl3175-bib-0091] Stern, D. L. , and V. Orgogozo . 2008 The loci of evolution: how predictable is genetic evolution? Evolution 62:2155–2177. 18616572 10.1111/j.1558-5646.2008.00450.x PMC2613234

[evl3175-bib-0092] Terekhanova, N. V. , M. D. Logacheva , A. A. Penin , T. V. Neretina , A. E. Barmintseva , G. A. Bazykin , et al. 2014 Fast evolution from precast bricks: genomics of young freshwater populations of threespine stickleback *Gasterosteus aculeatus* . PLoS Genet. 10:e1004696. 25299485 10.1371/journal.pgen.1004696 PMC4191950

[evl3175-bib-0093] Tuttle, E M. , A O. Bergland , M L. Korody , M S. Brewer , D J. Newhouse , P. Minx , et al. 2016 Divergence and functional degradation of a sex chromosome‐like supergene. Curr. Biol. 26:344–350. 26804558 10.1016/j.cub.2015.11.069 PMC4747794

[evl3175-bib-0094] Villablanca, E. J. , A. Renucci , D. Sapède , V. Lec , F. Soubiran , P. C. Sandoval , et al. 2006 Control of cell migration in the zebrafish lateral line: implication of the gene “tumour‐associated calcium signal transducer,” tacstd. Dev. Dyn. 235:1578–1588. 16552761 10.1002/dvdy.20743

[evl3175-bib-0095] Wada, H. , A. Ghysen , C. Satou , S.‐i. Higashijima , K. Kawakami , S. Hamaguchi , et al. 2010 Dermal morphogenesis controls lateral line patterning during postembryonic development of teleost fish. Dev. Biol. 340:583–594. 20171200 10.1016/j.ydbio.2010.02.017

[evl3175-bib-0096] Wada, H. , C. Dambly‐Chaudière , K. Kawakami , and A. Ghysen . 2013 Innervation is required for sense organ development in the lateral line system of adult zebrafish. Proc. Natl. Acad. Sci. USA 110:5659–5664. 23509277 10.1073/pnas.1214004110 PMC3619376

[evl3175-bib-0097] Wada, H. , M. Iwasaki , and K. Kawakami . 2014 Development of the lateral line canal system through a bone remodeling process in zebrafish. Dev. Biol. 392:1–14. 24836859 10.1016/j.ydbio.2014.05.004

[evl3175-bib-0098] Wagner, G. P. , J. P. Kenney‐Hunt , M. Pavlicev , J. R. Peck , D. Waxman , and J. M. Cheverud . 2008 Pleiotropic scaling of gene effects and the ‘cost of complexity’. Nature 452:470–472. 18368117 10.1038/nature06756

[evl3175-bib-0099] Wallbank, R. W. R. , S. W. Baxter , C. Pardo‐Diaz , J. J. Hanly , S. H. Martin , J. Mallet , et al. 2016 Evolutionary novelty in a butterfly wing pattern through enhancer shuffling. PLoS Biol. 14:e1002353. 26771987 10.1371/journal.pbio.1002353 PMC4714872

[evl3175-bib-0100] Wang, J. , Y. Wurm , M. Nipitwattanaphon , O. Riba‐Grognuz , Y. ‐ C. Huang , D. Shoemaker , et al. 2013 A Y‐like social chromosome causes alternative colony organization in fire ants. Nature 493:664–668. 23334415 10.1038/nature11832

[evl3175-bib-0101] Wang, Z. , B. Y. Liao , and J. Zhang . 2010 Genomic patterns of pleiotropy and the evolution of complexity. Proc. Natl. Acad. Sci. USA 107:18034–18039. 20876104 10.1073/pnas.1004666107 PMC2964231

[evl3175-bib-0102] Wark, A. R. , M. G. Mills , L. H. Dang , Y. F. Chan , F. C. Jones , S. D. Brady , et al. 2012 Genetic architecture of variation in the lateral line sensory system of threespine sticklebacks. G3 2:1047–1056. 22973542 10.1534/g3.112.003079 PMC3429919

[evl3175-bib-0103] Wark, A. R. , and C. L. Peichel . 2010 Lateral line diversity among ecologically divergent threespine stickleback populations. J. Exp. Biol. 213:108–117. 20008367 10.1242/jeb.031625 PMC2792588

[evl3175-bib-0104] Watanabe, K. , S. Stringer , O. Frei , M. Umićević Mirkov , C. de Leeuw , T. J. C. Polderman , et al. 2019 A global overview of pleiotropy and genetic architecture in complex traits. Nat. Genet. 51:1339–1348. 31427789 10.1038/s41588-019-0481-0

[evl3175-bib-0105] Westram, A. M. , M. Rafajlović , P. Chaube , R. Faria , T. Larsson , M. Panova , et al. 2018 Clines on the seashore: the genomic architecture underlying rapid divergence in the face of gene flow. Evol. Lett. 2:297–309. 30283683 10.1002/evl3.74 PMC6121805

[evl3175-bib-0106] Wucherpfennig, J. I. , C. T. Miller , and D. M. Kingsley . 2019 Efficient CRISPR‐Cas 9 editing of major evolutionary loci in sticklebacks. Evol. Ecol. Res. 20:107–132. PMC8664273 34899072

[evl3175-bib-0107] Yamasaki, Y. Y. , S. Mori , T. Kokita , and J. Kitano . 2019 Armour plate diversity in Japanese freshwater threespine stickleback (*Gasterosteus aculeatus*). Evol. Ecol. Res. 20:51–67. 10.1002/ece3.10077 PMC10191778 37206690

[evl3175-bib-0108] Yeaman, S. , and M. C. Whitlock . 2011 The genetic architecture of adaptation under migration‐selection balance. Evolution 65:1897–1911. 21729046 10.1111/j.1558-5646.2011.01269.x

[evl3175-bib-0109] Yoshizawa, M. , Y. Yamamoto , K. E. O'Quin , and W. R. Jeffery . 2012 Evolution of an adaptive behavior and its sensory receptors promotes eye regression in blind cavefish. BMC Biol. 10:108. 23270452 10.1186/1741-7007-10-108 PMC3565949

