# Supplementary material for: Adaptation via pleiotropy and linkage: Association mapping reveals a complex genetic architecture within the stickleback Eda locus
Source: Evol Lett. 2020 May 27;4(4):282–301. doi: 10.1002/evl3.175 (PMC7403726; doi:10.1002/evl3.175)
Supplement: Supplementary file 1 — Figure S1. Twelve neuromast lateral lines were phenotyped using either DASPEI or alkaline phosphatase staining to identify and count neuromasts. Figure S2. Landmark positions and descriptions used for geometric morphometrics. Figure S3. Visual genotypes of Lake Washington fish reveal historical recombination events and frequency of NAKA SNP. Figure S4. Association mapping of geometric morphometric traits in Lake Washington stickleback reveals three traits that are associated with the Eda haplotype. Figure S5. NAKA SNP near Eda is conserved across most percomorph species and likely lost in marine stickleback. Figure S6. Recombination breakpoints in low‐plated Lake Washington fish narrows the region containing the causative mutation to 1400 base pairs. Figure S7. Gill raker length maps to SNPs12/13 in Puget Sound. Table S1. Summary of genotyping assays used in this study. Table S2. Summary of mapping results performed in this study. Table S3. Estimated effects of Cnv770 and SNPs12/13 on traits that map to the haplotype. [file EVL3-4-282-s001.pdf]

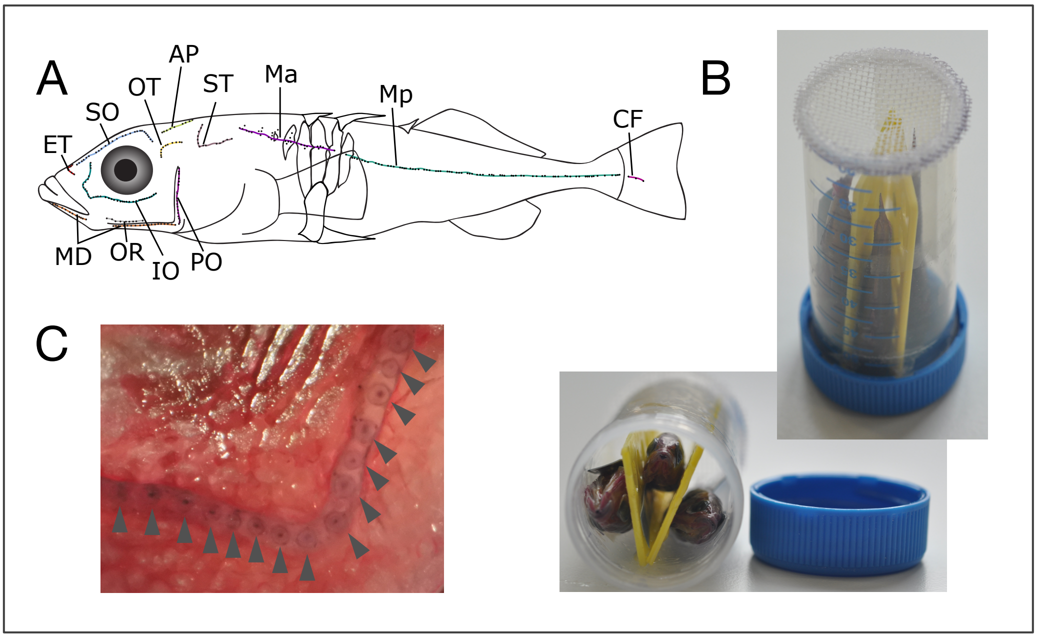

Figure S1. Twelve neuromast lateral lines were phenotyped using either DASPEI or alkaline phosphatase staining to identify and count neuromasts. (A) Lateral lines were identified following Wark and Peichel (2010). The twelve lines are: AP-anterior pit; CF-caudal fin; ET-ethmoid; IO-infraorbital; Ma-anterior main trunk; MD-mandibular; Mp-posterior main trunk; OR-oral; OT-otic; PO-preopercular; SO-supraorbital; ST-supratemporal. (B) Fish were stained with alkaline phosphatase in custom staining chambers made from 50mL conical tubes, nylon mesh and pipette tip refill wafers. These chambers fit 3 fish at a time with their ID tags, and washes were easily exchanged through the mesh without opening the tubes. (C) A representative photo of alkaline phosphatase staining of neuromasts (arrowheads) and Alizarin red staining of bone at the intersection of the PO and MD lines.

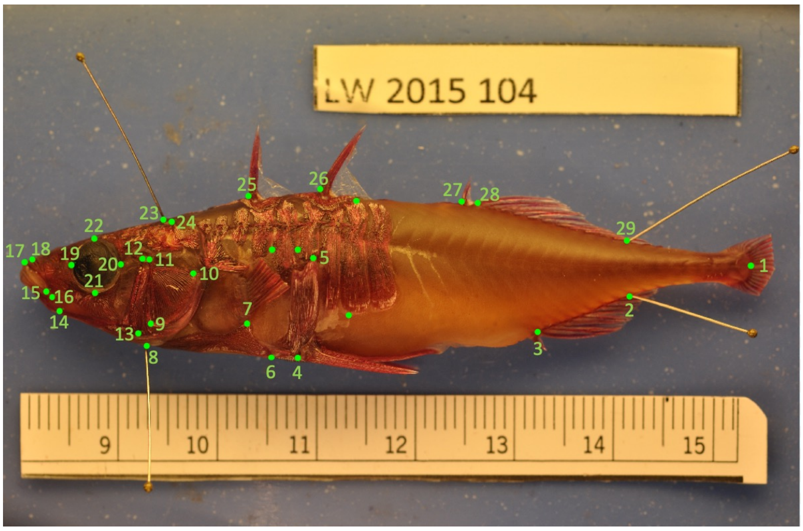

Landmark	Description
1	Posterior extent of caudal peduncle
2	Posterior insertion of anal fin
3	Anterior insertion of anal fin
4	Insertion point of pelvic spine into the pelvic girdle
5	Dorsal extent of the ascending branch of the pelvis
6	Posterior extent of ectocoracoid
7	Dorsal extent of ectocoracoid
8	Anterior extent of ectocoracoid
9	Ventral extent of operculum
10	Posteriodorsal extent of operculum
11	Anteriodorsal extent of operculum
12	Dorsal extent of preopercular
13	Posterioventral extent of preopercular
14	Anterioventral extent of preopercular
15	Posterior extent of premaxilla
16	Posterior extent of maxilla
17	Anterior-most extent of the premaxilla
18	Anterior extent of maxilla
19	Anterior extent of orbit
20	Posterior extent of orbit
21	Ventral extent of orbit
22	Dorsal margin of the orbit in line with the eye’s midpoint
23	Supraoccipital notch lateral to the dorsal midline
24	Posterior extent of supraoccipital
25	Anterior insertion of first dorsal spine
26	Anterior insertion of second dorsal spine
27	Anterior insertion of third dorsal spine
28	Anterior insertion of the dorsal fin at the first soft ray
29	Posterior insertion of dorsal fin at the first soft ray

Figure S2. Landmark positions and descriptions used for geometric morphometrics. Landmarks were chosen based on previous QTL mapping studies between marine and freshwater fish (Table S2).

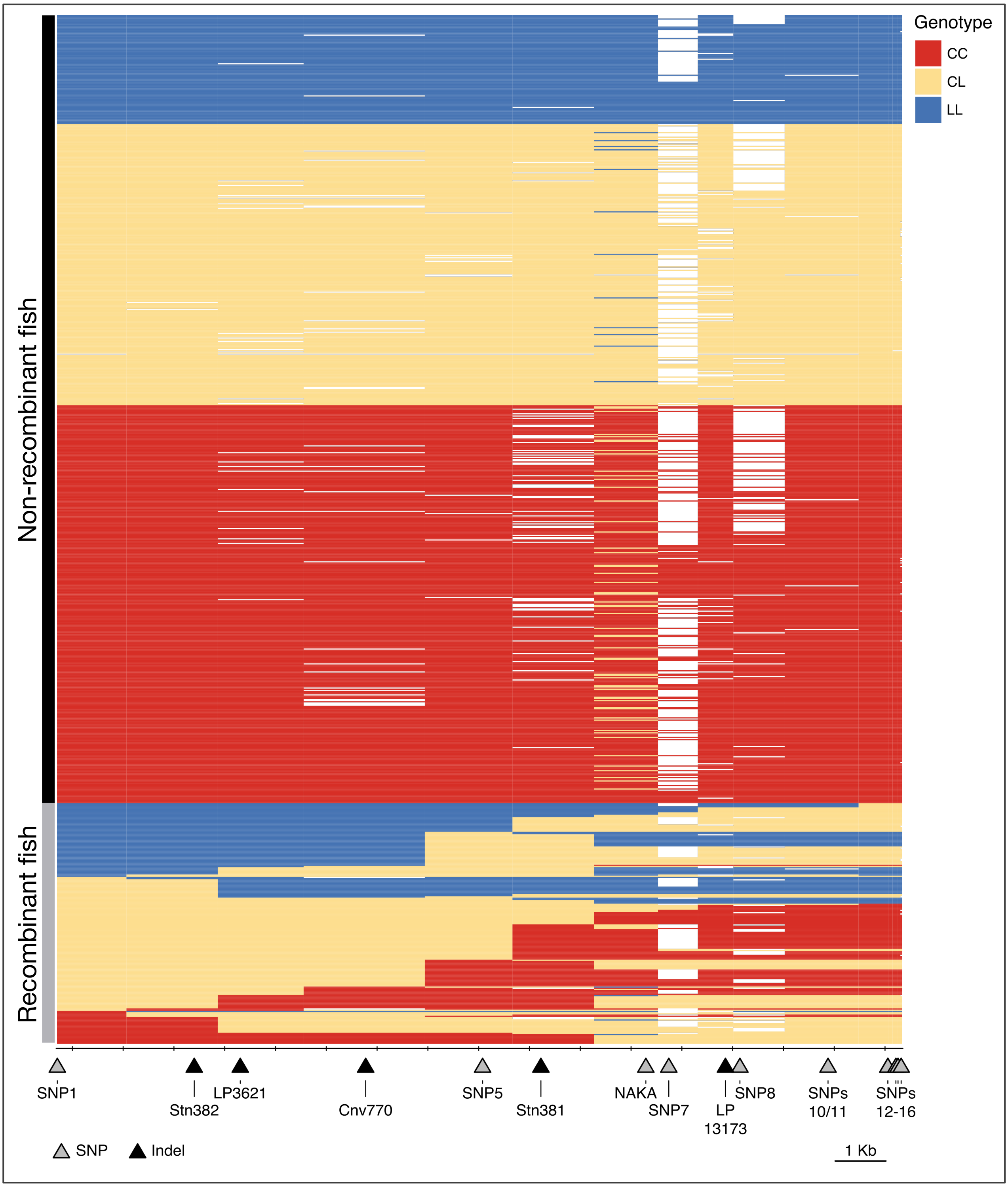
Genotype
CC
CL
LL
Non-recombinant fish
Recombinant fish
SNP1
Stn382
LP3621
Cnv770
SNP5
Stn381
NAKA
SNP7
LP
13173
SNP8
SNPs
10/11
SNPs
12-16
SNP
Indel
1 Kb

Figure S3. Visual genotypes of Lake Washington fish reveal historical recombination events and frequency of NAKA SNP. Wild-caught adult stickleback from Lake Washington (n = 885) were genotyped at a subset of 16 SNPs or indels across the haplotype and SNP1. Most fish showed no evidence of recombination within the 16 kb haplotype (top). However, we did find evidence of at least one historical recombination event within the haplotype in 198, or 22%, of the fish (bottom, same as Figure 5). In addition to visualizing recombination breakpoints, this figure highlights the frequency of the "L" allele of the NAKA SNP on an otherwise "C" haplotype. For example, we see complete correspondence between NAKA and the other genotypes within the LL non-recombinant fish (blue, top). However, within the generally CL non-recombinant fish, there are 12 blue LL genotypes at NAKA (n = 12 of 244 C haplotypes, 4.9%). Within the generally CC non-recombinant fish there are 56 fish CL at NAKA (n = 56 of 694 C haplotypes, 8.1%). The frequencies of the L allele at the NAKA SNP on generally C haplotype is 9.4% in Puget Sound (Figure 2) and 7.2% in Lake Washington. The markers are depicted as triangles at their physical location within the haplotype and labelled at the bottom of the figure. Tick marks start at the beginning of intron 1 of *Eda* (the presumed start of the haplotype) and are spaced every 1000 bases for scale. Genotypes are represented visually as CC (homozygous for the completely-plated allele), CL (heterozygous) or LL (homozygous for the low-plated, typically freshwater, allele).

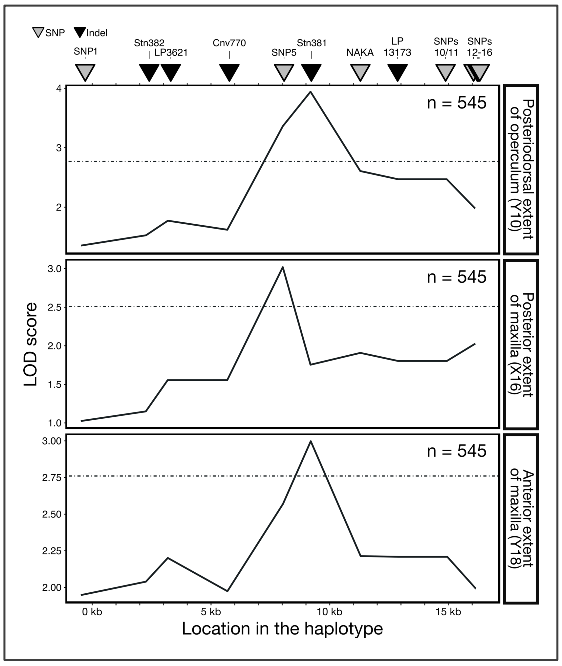

Figure S4. Association mapping of geometric morphometric traits in Lake Washington stickleback reveals three traits that are associated with the *Eda* haplotype. The strength of association between each marker and each phenotype was calculated as a log odds likelihood (LOD) score compared with the model of no association between marker and phenotype. These LOD curves are plotted for three geometric morphometric landmarks that are significant following correction for multiple comparisons (Figure S2). Posteriodorsal extent of the operculum (Y10) and anterior extent of maxilla (Y18) map most strongly to Stn381. The posterior extent of the maxilla (X16) maps most strongly to SNP5. These patterns of association are consistent with either separate, linked causative mutations, or a single pleiotropic mutation between SNP5 and Stn381. The dashed lines represent the LOD threshold.

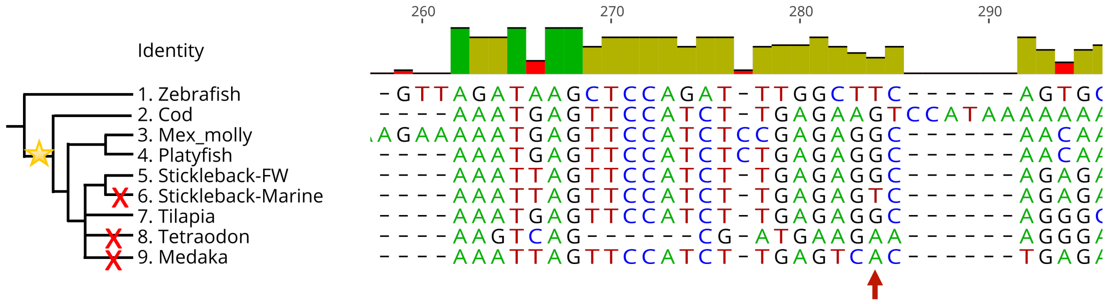

Figure S5. NAKA SNP near *Eda* is conserved across most percomorph species and likely lost in marine stickleback. Alignment of intergenic region downstream of Eda. Downstream genomic sequences were retrieved from NCBI using the stickleback Eda sequence as a query. Sequences were aligned using VISTA and corrected at the locus by eye. Zebrafish and cod are not members of the percomorph clade, yet cod shares a "G" at the NAKA SNP (red arrow) with other percomorphs (including freshwater, low-plated stickleback), suggesting that the NAKA SNP arose in the common ancestor of cod and percomorphs (yellow star). Three percomorphs do not have a "G" at the NAKA SNP position, suggesting it was lost in marine stickleback, tetraodon and medaka (red X's).

A

	Left plate count	SNP1	Stn382	LP3621	Cnv770	SNP5	Stn381	NAKA	SNP7	LP13173	SNP8	SNPs10/11	SNP12	SNP13	SNP14	SNP15	SNP16
*	7	CL	CL	LL	LL	LL	LL	LL	-	LL	LL	LL	LL	LL	LL	LL	LL
*	7	CL	CL	LL	LL	LL	LL	LL	LL	LL	LL	LL	LL	LL	LL	LL	LL
*	7	CL	CL	LL	LL	LL	LL	LL	-	LL	LL	LL	LL	LL	LL	LL	LL
	7	LL	LL	LL	LL	LL	LL	LL	-	LL	LL	LL	CL	CL	CL	CL	CL
	7	CL	CL	LL	LL	LL	LL	LL	LL	LL	LL	LL	LL	LL	LL	LL	LL
→	7	LL	LL	LL	CL	CL	CL	CL	CL	-	CL	CL	CL	CL	CL	CL	CL
	7	LL	LL	LL	LL	CL	CL	LL	LL	LL	LL	LL	LL	LL	LL	LL	LL
	7	CL	LL	LL	-	LL	LL	LL	LL	LL	LL	LL	LL	LL	LL	LL	LL
	7	LL	LL	LL	LL	LL	LL	LL	LL	LL	LL	LL	CL	CL	CL	CL	CL
	7	CC	LL	LL	LL	LL	LL	LL	-	-	LL	LL	LL	LL	LL	LL	LL
	7	LL	LL	LL	LL	LL	LL	LL	-	LL	LL	LL	CL	CL	CL	CL	CL
	7	LL	LL	LL	LL	CL	CL	CL	-	CL	CL	CL	CL	CL	CL	CL	CL
	8	LL	LL	LL	LL	LL	LL	LL	CL	CL	CL	CL	CL	CL	CL	CL	CL
	7	LL	LL	LL	LL	LL	LL	LL	LL	CL	CL	CL	CL	CL	CL	CL	CL
	7	LL	LL	LL	LL	LL	LL	LL	LL	CL	CL	CL	CL	CL	CL	CL	CL
	8	LL	LL	LL	LL	LL	CL	CL	-	CL	-	CL	CL	CL	CL	CL	CL
	7	CL	CL	LL	LL	LL	LL	LL	-	LL	-	LL	LL	LL	LL	LL	LL
	8	CL	LL	LL	LL	LL	LL	LL	-	LL	LL	LL	LL	LL	LL	LL	LL
	8	LL	LL	LL	LL	LL	LL	LL	-	LL	LL	LL	CL	CL	CL	CL	CL
	8	LL	LL	LL	LL	LL	LL	LL	CL	CL	CL	CL	CL	CL	CL	CL	CL
	8	LL	LL	LL	LL	CL	CL	LL	LL	LL	LL	LL	LL	LL	LL	LL	LL
	8	LL	LL	LL	LL	LL	CL	CL	CL	CL	CL	CL	CL	CL	CL	CL	-
	8	LL	LL	LL	LL	LL	LL	CL	CL	CL	CL	CL	CL	CL	CL	CL	CL
*	8	CL	CL	LL	LL	LL	LL	LL	LL	LL	LL	LL	LL	LL	LL	LL	LL
	9	LL	LL	LL	LL	CL	CL	CL	-	CL	-	CL	CL	CL	CL	CL	CL
	9	LL	LL	LL	LL	CL	CL	LL	LL	LL	LL	LL	LL	LL	LL	LL	LL
*	8	CL	CL	LL	LL	LL	LL	LL	LL	LL	LL	LL	LL	LL	LL	LL	LL
	9	LL	LL	LL	LL	LL	LL	LL	LL	CL	CL	CL	CL	CL	CL	CL	CL
	9	LL	LL	LL	LL	CL	CL	LL	LL	LL	LL	LL	LL	LL	LL	LL	LL
	10	LL	LL	LL	LL	CL	CL	LL	LL	LL	LL	LL	LL	LL	LL	LL	LL
	9	CL	CL	LL	LL	LL	LL	LL	-	LL	LL	LL	LL	LL	LL	LL	LL
	10	LL	LL	LL	LL	CL	CL	LL	LL	LL	LL	LL	LL	LL	LL	LL	LL
	10	LL	LL	LL	LL	LL	CL	CL	-	CL	CL	CL	CL	CL	CL	CL	CL
	11	LL	LL	LL	LL	LL	CL	CL	-	CL	CL	CL	CL	CL	CL	CL	CL
	10	LL	LL	LL	LL	LL	CL	CL	-	CL	CL	CL	CL	CL	CL	CL	CL
#	7	LL	LL	CL	CL	CL	CL	LL	LL	LL	LL	LL	LL	LL	LL	LL	LL
#	8	LL	LL	CL	CL	CL	CL	LL	LL	LL	LL	LL	LL	LL	LL	LL	LL
#	8	LL	LL	CL	CL	CL	CL	LL	LL	-	LL	LL	LL	LL	LL	LL	LL
	9	LL	LL	CL	CL	CL	CL	LL	LL	LL	LL	-	LL	LL	LL	LL	LL
#	11	LL	LL	CL	CL	CL	CL	LL	LL	LL	LL	LL	LL	LL	LL	LL	LL
	10	LL	LL	CL	CL	CL	CL	LL	LL	LL	LL	LL	LL	LL	LL	LL	LL

B

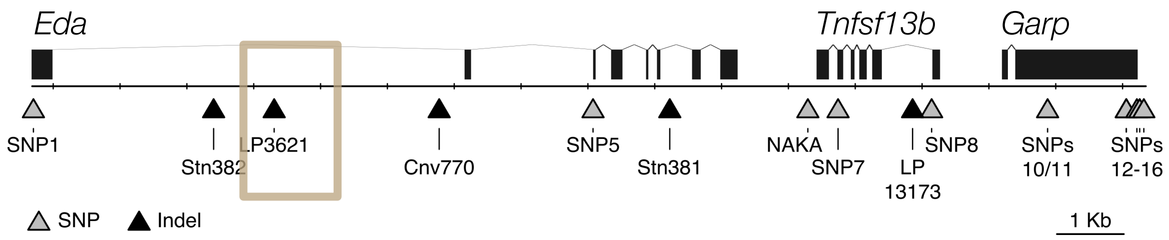

Figure S6. Recombination breakpoints in low-plated Lake Washington fish narrows the region containing the causative mutation to 1400 base pairs. Genotypes of low-plated fish at the 16 screened markers are shown for fish with evidence of at least one historical recombination. Together, these genotypes suggest that the causative allele is located between Stn382 and LP3621. Cloning and sequencing of five fish an asterisk next to their plate count identified the 5' recombination breakpoint at base pair 12,803,450 of the gasAcu1 genome. Including the sequences of four fish with a pound sign next to their plate count found no mutation for which these fish all carried two copies of the freshwater allele. We therefore conclude that the six fish on the bottom of the table may carry a unique set of marine alleles and refocused on the region between LP3621 and Cnv770, since all other low-plated fish are homozygous for the freshwater allele at LP3621. A single fish (arrow) recombines between LP3621 and Cnv770. This recombination breakpoint was mapped to base pair 12,804,851 in the gasAcu1 genome by cloning the recombinant allele. Therefore, the causative mutation lies within a region of 1,401 base pairs, marked with a box in (B).

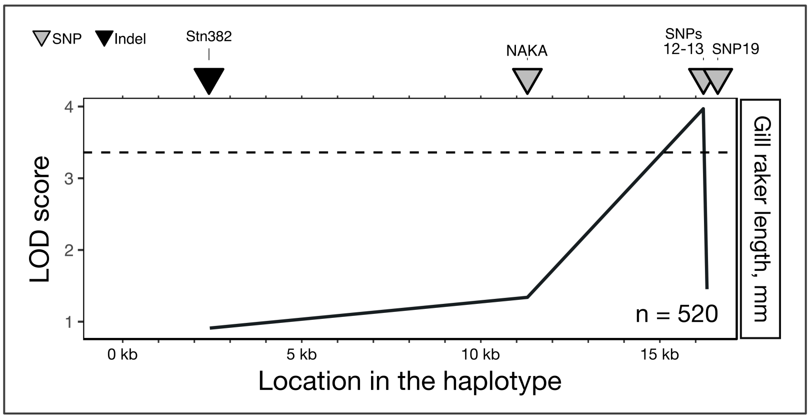

Figure S7. Gill raker length maps to SNPs12/13 in Puget Sound. Gill raker length was significantly associated with SNPs12/13 in the Puget Sound crosses when only Stn382 and SNPs12/13 were considered. In Lake Washington, the strongest signal of association between genotype and gill raker length was at the NAKA SNP (Figure 6). To assess the association between the NAKA SNP and gill raker length in Puget Sound, we genotyped the F1 offspring at the NAKA SNP, which was variable in two of the crosses, and reran the analysis. This confirmed the first analysis- the association of gill raker length and genotype is driven by the genotype at SNPs12/13 in Puget Sound, not the NAKA SNP or something outside of the haplotype (SNP19). The dashed line represents the LOD threshold.

Marker name	Description	Location on chr.IV of Broad/gasAcu1 genome assembly	Location in haplotype‡	Forward primer (5' - 3')	Reverse primer (5' - 3')	Genotyping method	Restriction enzyme	Population(s) screened
Cnv767[1]	Intergenic FW insertion (68bp)	12,790,042-12,790,110	-	TGTTGTGCAGTCGGGTGCCAGT	GGCTTCCATTGTGCCTGGTGCCTT	PCR		PS / --
SNP1[2]	Nonsynonymous SNP in *Eda*	12,800,238	-	AGATGCCGAGGTAGAGAGCA	GTAGCAGACGAGCGTGACAG	Restriction digest	BtsCI	-- / LW
Stn382[2]	Intronic FW deletion (66bp)	12,802,847	2,400	CCCTTAGAGAATTTCCTAGCAG	CTTGTCCCGGATCATACGC	PCR		PS / LW
LP3621	Intronic FW deletion (16bp)	12,803,862	3,306	CCTGGTGGACGGATAGAGCA	ATGTTGCCTGTCAGCAGCCA	PCR		-- / LW
Cnv770[1]	Intronic FW insertion (107bp)	12,806,052-12,806,138	5,775	AGGCATCGCGCTCACGTTGA	TCGCCCGYTGAGTTATGCCCC	PCR		-- / LW
SNP5[2]	Nonsynonymous SNP in *Eda*	12,808,303	8,077	GTTCAGGAGAACGTTTCAAGCT	GCCGCTTTTTCCCTGTGAAG	Restriction digest	Taqa1	-- / LW
Stn381[2]	Intronic FW insertion (19bp)	12,809,414-12,809,601	9,225	CCGCTACACACGGACTTACA*	ATTCGAGGGTTCAGCTCTGG	PCR		-- / LW
NAKA.SNP[3]	Intergenic SNP	12,811,481	11,291	TTGGACACTGCTGGCACGGG	TCACACTCTGRTTAACCCCCGGA	Restriction digest	MnlI	-- / LW
SNP7[2]	Nonsynonymous SNP in *Tnfsf13b*	12,811,933	11,743	AGGATGTAGTCATCTTGGACATTGT	CTTCAGAGAACTCAGACTGTTTGTG	MAMA PCR - FW allele		-- / LW
				AGGATGTAGTCATCTTGGACATTGT	TCAGAGAACTCAGACTGTTTGCC	MAMA PCR - Marine allele		
LP13173	Intronic FW insertion (20bp)	12,813,043-12,813,066	12,858	TACGCTAGTTAGCTGCTGGC	AAATGAGGGAATGGGGCCTG	PCR		-- / LW
SNP8[2]	Synonymous SNP in *Tnfsf13b*	12,813,328	13,143	CAAACAGCTTCAGAGACTGTGC	CCTGAAGCAGCTGCACAAGA	MAMA PCR - FW allele		-- / LW
				CAAACAGCTTCAGAGACTGTGC	CCTGAAGCAGCTGCACAAAG	MAMA PCR - Marine allele		
SNPs10/11[2]	Nonsynonymous SNP in *Garp*	12,815,024	14,878	ACGACTTGGGTGATGATGCA	TCAAGCCTGTACAACTGGTCA	Restriction digest	AluI	-- / LW
SNP12[2]	Nonsynonymous SNP in *Garp*	12,816,201	16,055	TCTGCTACCAAGCAGTTTGA	AGTTTGCTATTCAGTTTCTCAC	Sequencing in LW	Mfe-HF in PS	PS / LW
SNP13[2]	Synonymous SNP in *Garp*	12,816,202	16,056	TCTGCTACCAAGCAGTTTGA	AGTTTGCTATTCAGTTTCTCAC	Sequencing in LW	Mfe-HF in PS	PS / LW
SNP14[2]	Nonsynonymous SNP in *Garp*	12,816,360	16,214	TCTGCTACCAAGCAGTTTGA	AGTTTGCTATTCAGTTTCTCAC	Sequencing		-- / LW
SNP15[2]	SNP in *Garp* 3' UTR	12,816,402	16,256	TCTGCTACCAAGCAGTTTGA	AGTTTGCTATTCAGTTTCTCAC	Sequencing		-- / LW
SNP16[2]	SNP in *Garp* 3' UTR	12,816,464	16,318	TCTGCTACCAAGCAGTTTGA	AGTTTGCTATTCAGTTTCTCAC	Sequencing		-- / LW
SNP19[2]	Nonsynonymous SNP in *Gjb1*	12,826,854	-	CAGCTCATCCTGGTCTCCAC	TCCGGTGATCTGGAACTTCT	Restriction digest	Eco0109I	PS / --
IDH[4]	Y-chr. deletion in 3'UTR of *Idh*	-	-	GGGACGAGCAAGATTTATTG	TTATCGTTAGCCAGGAGATGG	PCR		PS / LW

[1]Lowe *et al.* 2018; [2]Colosimo et al. 2005; [3]O'Brown et al. 2015; [4]Peichel et al. 2004
*Redesigned primer; previously published primer sat on a polymorphic site in Lake Washington
‡Beginning of haplotype is set at start of *Eda* intron 1

Table S1. Summary of genotyping assays used in this study. PCR-based assays were used to genotype fish at the listed SNPs and indels. Information on the markers and the PCR-based assays used to genotype fish at each marker are listed. PCR = length polymorphism analysis; MAMA PCR = mismatch amplification mutation assay; PS = Puget Sound crosses; LW = Lake Washington wild-caught adults.

Table S2. Summary of mapping results performed in this study. The marker with the highest LOD score for each trait is listed in each mapping population with the corresponding LOD score, the covariates used in the analysis and the PVE explained by the listed marker. The p-value listed for each trait and marker combination is calculated from 5000 permutations of the data and represent the proportion of permutations that have an equal or higher LOD score at that marker. The p-value threshold was adjusted using a Bonferroni correction for the number of traits tested for each mapping population. PS = Puget Sound crosses; LW = Lake Washington wild-caught adults; NAKA = Puget Sound crosses between fish heterozygous at the NAKA SNP only; PVE = percent variation explained. This table is provided as a separate Excel file.

				Cnv770 CC / SNPs 12/13 CC	Cnv770 CC / SNPs 12/13 CL	Cnv770 CL / SNPs 12/13 CC	Cnv770 CL / SNPs 12/13 CL	Cnv770 CL / SNPs 12/13 LL	Cnv770 LL / SNPs 12/13 CC	Cnv770 LL / SNPs 12/13 CL	Cnv770 LL / SNPs 12/13 LL
Mapping population	Trait type	Trait name	n	trait value ± s.d.	trait value ± s.d.	trait value ± s.d.	trait value ± s.d.	trait value ± s.d.	trait value ± s.d.	trait value ± s.d.	trait value ± s.d.
Puget Sound crosses	Lateral line	Mp (posterior main trunk line) neuromasts per segment	497	2.37 ± 0.04		2.10 ± 0.05	2.31 ± 0.03		1.78 ± 0.12	1.44 ± 0.06	1.32 ± 0.06
	Lateral line	Dorsal-Ventral (D-V) neuromast patterning	498	0.99 ± 0.01		0.94 ± 0.01	0.96 ± 0.01		0.35 ± 0.03	0.22 ± 0.01	0.08 ± 0.01
	Meristic	Left plate count	578	34.1 ± 0.2		33.3 ± 0.3	33.3 ± 0.2		17.0 ± 0.8	13.3 ± 0.3	9.0 ± 0.3
	Metric	Middle gill raker length	571	1.64 ± 0.01		1.69 ± 0.02	1.64 ± 0.01		1.70 ± 0.04	1.66 ± 0.02	1.56 ± 0.02
Lake Washington wild-caught	Lateral line	Mp (posterior main trunk line) neuromasts per segment	460	2.20 ± 0.05	1.95 ± 0.13	1.90 ± 0.08	1.87 ± 0.05	1.83 ± 0.21	2.08 ± 0.41	1.55 ± 0.09	1.45 ± 0.06
	Lateral line	Dorsal-Ventral (D-V) neuromast patterning	460	0.93 ± 0.02	0.80 ± 0.06	0.74 ± 0.04	0.66 ± 0.02	0.32 ± 0.10	0.65 ± 0.18	0.16 ± 0.04	0.09 ± 0.03
	Meristic	Left plate count	696	33.2 ± 0.4	29.9 ± 0.9	27.7 ± 0.6	26.1 ± 0.4	12.0 ± 1.3	21.9 ± 3.9	12.1 ± 0.7	8.5 ± 0.4
	Morphometric	Posteriodorsal extent of operculum (Y10)	545	0.013 ± 0.001	0.012 ± 0.001	0.012 ± 0.001	0.013 ± 0.001	0.011 ± 0.002	0.021 ± 0.005	0.012 ± 0.001	0.011 ± 0.001
	Morphometric	Posterior extent of maxilla (X16)	545	-0.185 ± 0.000	-0.186 ± 0.001	-0.185 ± 0.000	-0.185 ± 0.000	-0.187 ± 0.001	-0.185 ± 0.003	-0.186 ± 0.001	-0.186 ± 0.000
	Morphometric	Anterior extent of maxilla (Y18)	545	0.013 ± 0.001	0.014 ± 0.001	0.013 ± 0.001	0.013 ± 0.001	0.016 ± 0.003	0.005 ± 0.006	0.015 ± 0.001	0.015 ± 0.001

Table S3. Estimated effects of Cnv770 and SNPs12/13 on traits that map to the haplotype. The R package *emmeans* was used to estimated the marginal mean trait values and standard deviation for fish with the specific combination of genotypes at Cnv770 and SNPs12/13. The linear model used included the covariates used in trait mapping. Figures 3 and 7 depict the data visually, though the points plotted are adjusted trait values, whereas the numbers represented here are estimates for real trait values.
